# ABX-1431 inhibits the development of endometrial adenocarcinoma and reverses progesterone resistance by targeting MGLL

**DOI:** 10.1038/s41419-022-05507-z

**Published:** 2022-12-23

**Authors:** Xiaohong Ma, Min Xia, Lina Wei, Kui Guo, Rui Sun, Yao Liu, Chunping Qiu, Jie Jiang

**Affiliations:** 1 Department of Gynecology and Obstetrics, Qilu Hospital of Shandong University, 250012 Jinan, China; 2 Department of Gynecology and Obstetrics, The Affiliated Yantai Yuhuangding Hospital of Qingdao University, 264000 Yantai, China; 3 Gynecologic Oncology Key Laboratory of Shandong Province, Qilu Hospital of Shandong University, 250012 Jinan, China

**Keywords:** Cancer therapeutic resistance, Targeted therapies

## Abstract

Endometrial cancer is a common gynecological malignancy. With the onset of EC patients younger, conservative treatment with progesterone has become an important option for patients trying to preserve reproductive function. However, progesterone resistance is a key factor affecting the efficacy of therapy and it is urgent to clarify the mechanism so as to propose a potential target and inhibit the development of endometrial adenocarcinoma and progesterone resistance. MGLL, an important factor involved in lipid mobilization, is overexpressed in many tumors, however the biological function of MGLL in the development of endometrial adenocarcinoma and the process of progesterone resistance still remains unclear. In this study, we first found MGLL was highly expressed in progesterone resistant samples of endometrial adenocarcinoma, and then we verified its expression was increased in endometrial adenocarcinoma. Through in vitro and in vivo experiments, we demonstrated that overexpression of MGLL promoted tumor proliferation, metastasis and the occurrence of progestogen resistance, knockdown MGLL inhibited tumor proliferation, metastasis and reversed progestogen resistance. In addition, knockdown of MGLL can sensitize endometrial adenocarcinoma cells to progesterone, possibly by affecting ROS generation and reducing the expression of AKR1C1. Finally, it was verified that ABX-1431, MGLL inhibitor, reversed progesterone resistance and enhanced the sensitivity of endometrial adenocarcinoma to progesterone both in vitro and in vivo. In conclusion, the high expression of MGLL is involved in the occurrence and development of endometrial adenocarcinoma and progesterone resistance. Targeted inhibition of MGLL by inhibitors may be an effective method for the treatment of progesterone resistance in endometrial adenocarcinoma.

## Introduction

Endometrial carcinoma (EC) is one of the most common gynecologic malignant tumors, ranking in 4th and 6th place of all cancers with respect to morbidity and mortality, respectively [1]. Endometrioid adenocarcinoma (EAC) is mainly associated with obesity, hypertension and other factors, about 5% of patients with EAC develop the disease before the age of 40, but in recent years, statistics have found that the proportion of patients with the disease before the age of 40 has increased to 10%, these patients are normally inclined to select progesterone-based therapy. Studies revealed that approximately 55% of EAC patients showed a complete response to conservative treatment that included medroxy-progesterone acetate (MPA), with a 47% recurrence rate [2]. Nevertheless, other studies have shown that more than 30% of EAC patients do not respond to MPA and acquired resistance to progesterone during the treatment [3]. In consequence, progesterone resistance has become an urgent clinical problem to be solved. In this study, we deeply explored the molecular mechanism of progesterone resistance in EAC, hoping to improve progesterone resistance and poor prognosis of it.

Monoacylglycerol lipase (MGLL, also MAGL) is an enzyme belonging to the family of serine hydrolases that preferentially catalyses the hydrolysis of mono-triglycerides to glycerol and fatty acids and specializes in the degradation of endocannabinoids to the major enzymes responsible for controlling and regulating the levels of this cannabinoid [4, 5]. It has become more and more important as an integrated metabolic hub, which not only controls the level of 2-arachidonoylglycerol(2-AG), but also controls the level of other monoacylglycerides. In addition, it controls the free fatty acids produced by their hydrolysis as well as other levels with proinflammatory or tumorigenic effects from the further metabolism of fatty acids. Accumulating evidence suggests that MGLL promotes tumor invasion and metastasis by up-regulating tumorigenic signals [6]. In cancer cells, MGLL hydrolyses monoacylglycerols into free fatty acids, maintains elevated levels of these molecules, and further converts them into different tumorigenic lipids such as lysophosphatidic acid (LPA), prostaglandin E2 (PGE2) [7, 8]. It is well-known that EAC is a malignant tumor associated with abnormal lipid metabolism, and MGLL may play an important role in it, our group found that MGLL was overexpressed in progesterone resistance cells compared to sensitivity cells in previous studies. However, the function of MGLL in EAC and progesterone resistance.

ABX-1431 is an efficient inhibitor of MGLL which is identified and developed by Cisar et al. [9]. Studies have shown that ABX-1431 inhibit MGLL obviously with dose-dependent in vitro. At present, ABX-1431 has positive clinical results [10, 11], and MGLL inhibitors have good prospects for application in a variety of human diseases. However, there is no researches associated with ABX-1431 in EAC.

In the present study, we explored the function and mechanism of MGLL in the development of EAC and progesterone resistance by carrying out a range of experiments in vivo and in vitro. Meanwhile, we investigated the efficacy of ABX-1431 for reversing progesterone resistance so as to provide efficient treatment for clinic in EAC.

## Results

### MGLL is a key gene high expressed in EAC and correlated to the progesterone resistance

**Fig. 1:**
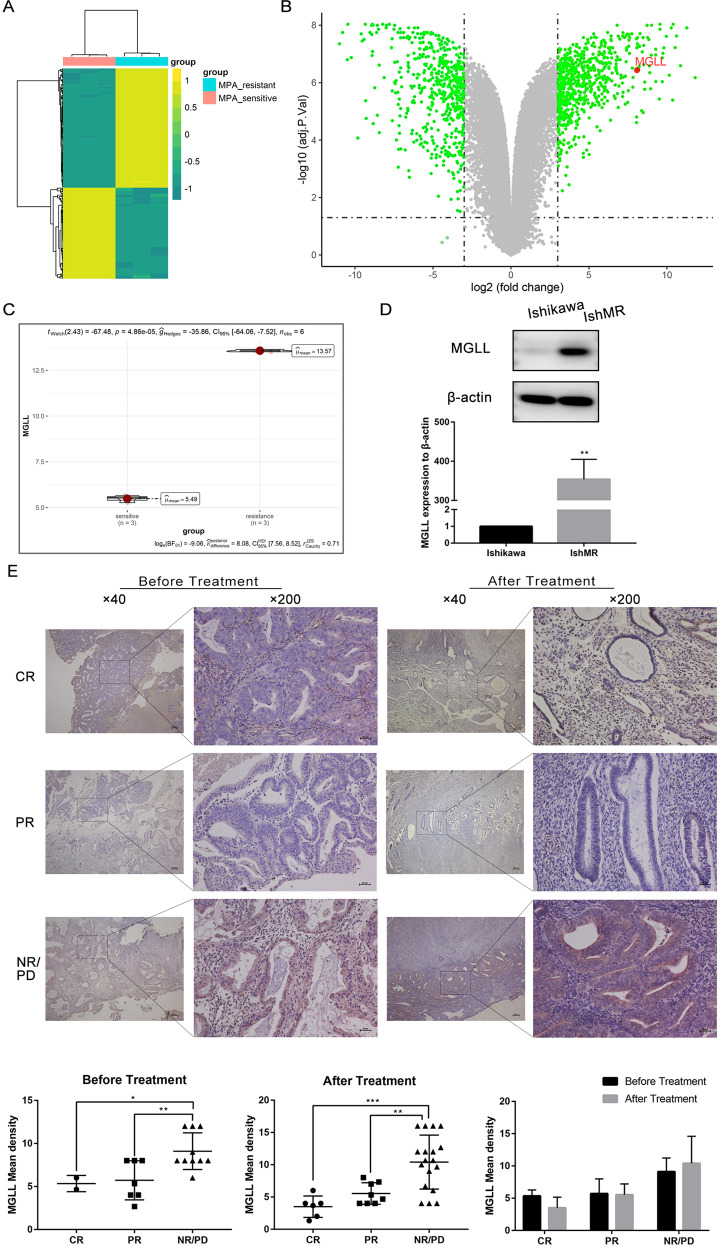

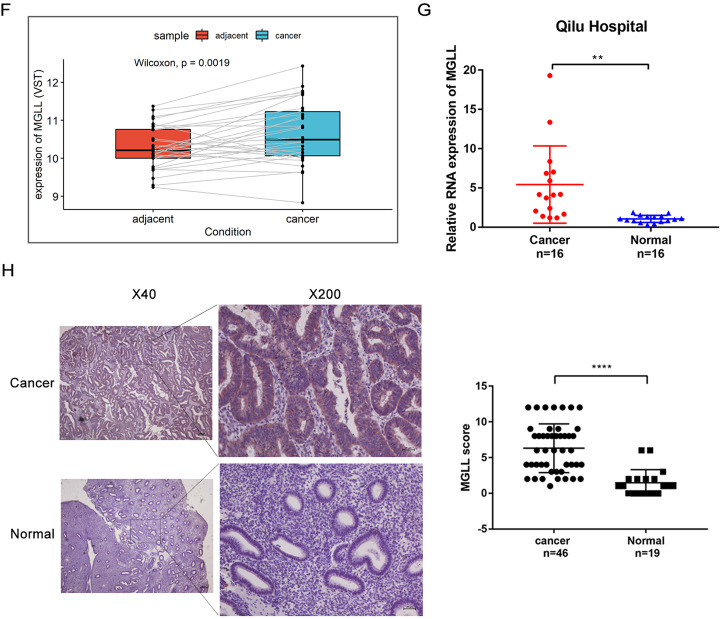
MGLL is a key gene high expressed in EAC and correlated to the progesterone resistance. **A** Hierarchically clustered heatmap of differentially expressed genes in IshMR and Ish cells. **B** Volcano plot of RNA-seq from microarray data in IshMR and Ish cells. The green dots represent DEGs based on a |FC | of ≥3. The red dots represent MGLL. **C** The violin plot depicting the expression distribution of MGLL between IshMR and Ish cells. **D** MGLL protein level in Ish and IshMR cells. **E** Representative images of IHC staining of MGLL in endometrium before and after progesterone treatment in CR, PR, and NR/PD group. MGLL IHC scores in endometrium before and after progesterone treatment were analyzed by Image-Pro Plus 6.0. The Score = (percentage of cells of weak intensity × 1) + (percentage of cells of moderate intensity × 2) + (percentage of cells of strong intensity × 3). **F** Validation of MGLL expression in EC and adjacent samples from GEO databases, GSE183185. The mRNA level of MGLL was analyzed with the “limma R” package using Student’s t test. **G** MGLL mRNA level in 16 EAC tissues and its adjacent tissues. **H** Representative images of IHC staining of MGLL in EAC and normal tissues. CR complete response, PR partial response, NR/PD no response/progression disease, Ish Ishikawa cells, IshMR MPA-resistant cell line of Ish cells. *P < 0.05, **P < 0.01, ***P < 0.001 and ****P < 0.0001 for statistical analysis of the indicated groups.

We analyzed the microarray data of EAC progesterone-sensitive cell line (Ishikawa, abbreviated as Ish) and the EAC progesterone-resistant cell line (IshikawaMR, abbreviated as IshMR), and the raw data arising from this analysis has been uploaded to the GEO database (reference: GSE121367). The threshold of differential genes was set at a adj.P < 0.005 and | fold change (FC)| ≥ 3, a heat map was created to show the differentially expressed genes (Fig. 1A). We found that the mRNA expression of MGLL in the IshMR cell line was higher than that in the Ish cell line (Fig. 1B, C). Subsequently, we performed Western blot and found that the expression of MGLL in IshMR cell line increased compared with Ish cell line (Fig. 1D and Original data 1A). We collected clinical specimens of endometrial adenocarcinoma/atypical hyperplasia from patients who treated with conservative progesterone for immunohistochemistry assay (IHC) experiments. According to clinical outcomes, the species were divided into three groups: complete response (CR), partial response (PR) and no response/progression disease (NR/PD). The staining of clinical specimens in the three groups before and after treatment showed that the expression of MGLL in the NR/PD group was significantly richer than that in the CR and PR groups(Fig. 1E). The expression of MGLL in CR and PR groups patients tended to decrease after progesterone treatment, while patients in the NR/PD group tended to increase, but the differences were not statistically significant.

To detect whether MGLL expression altered in EAC development, we further analyzed the data of GSE183185, a matched EAC and adjacent data in GEO database, and the results showed that MGLL expression was significantly higher in tumors compared with adjacent tissues (Fig. 1F). To verify the results from database, we analyzed MGLL mRNA level in 16 EAC/adjacent tissues, and the expression of MGLL was remarkedly up-regulated in EAC tissues compared to adjacent tissues (Fig. 1G). These findings were consistent with those from clinical samples (Fig. 1H).

In summary, MGLL is a key gene high expressed in EAC and correlated to the progesterone resistance.

### MGLL promotes the proliferation and inhibits the apoptosis of EAC cells

**Fig. 2:**
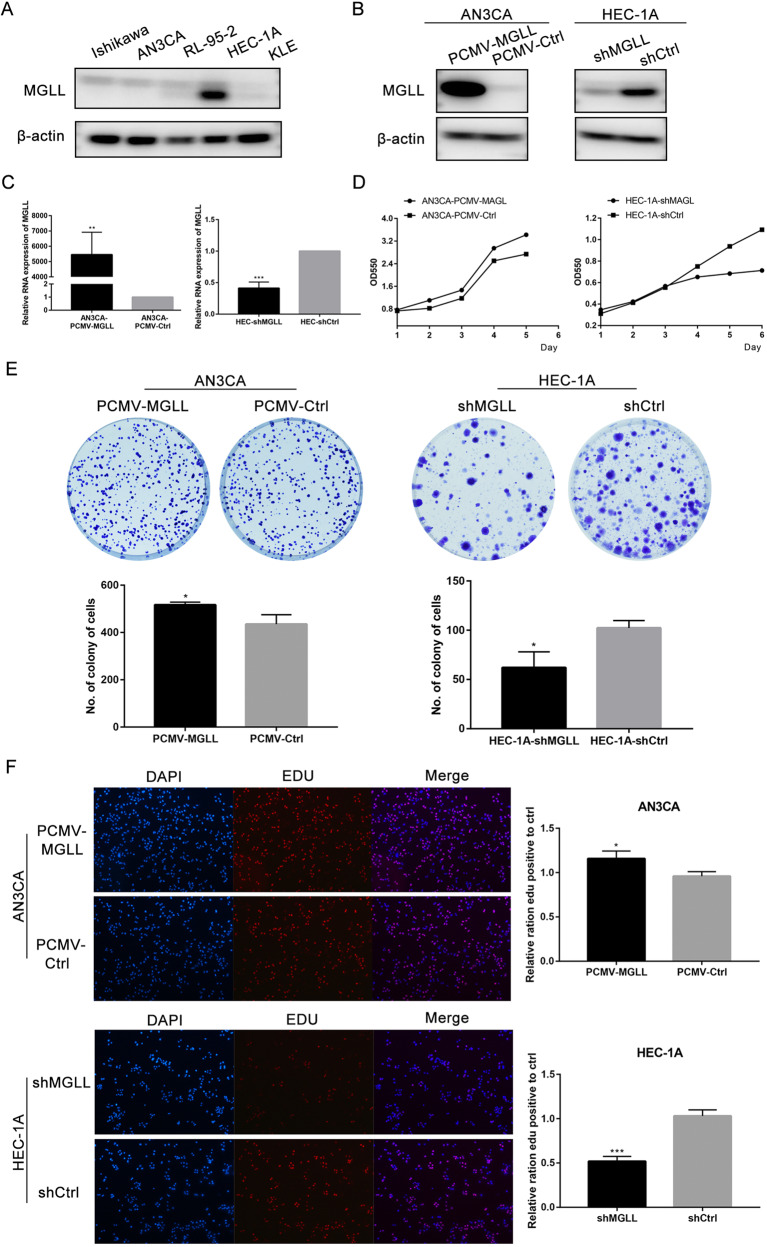

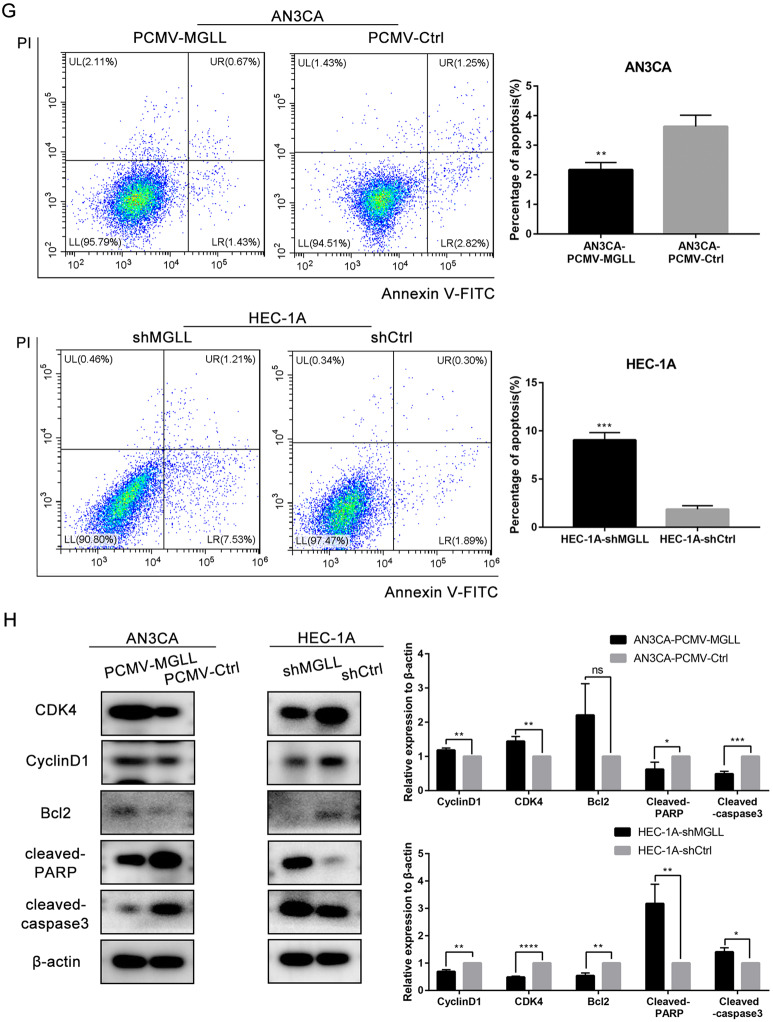
MGLL promotes the proliferation and inhibits the apoptosis of EAC cells. **A** Expression of MGLL in Ishikawa, AN3CA, RL95-2, HEC-1A and KLE cells. **B** Protein level of MGLL in AN3CA cells after MGLL knockdown and in HEC-1A cells after MGLL overexpressed. **C** mRNA level of MGLL in AN3CA cells after MGLL knockdown and in HEC-1A cells after MGLL overexpressed. **D** Growth curves of cells after MGLL deletion or overexpression compared with control cells. **E** The effect of MGLL overexpression and inhibition on colony formation. **F** Proliferation rates of cells overexpressing or knocking-out MGLL by EDU incorporation assay. **G** Proportion of apoptotic cells presented by apoptosis assay in cells after overexpression or knockdown of MGLL. **H** Expression of CDK4, CyclinD1, Bcl-2, cleaved-PARP and cleaved-caspase3 were determined by Western blot assay in EAC cells after overexpression or knockdown of MGLL. All experiments were repeated three times at least. *P < 0.05, **P < 0.01, ***P < 0.001 and ****P < 0.0001 for statistical analysis of the indicated groups.

We detected the expression of MGLL in 5 EAC cell lines and found that MGLL was barely expressed in AN3CA cells and abundant expressed in HEC-1A cells (Fig. 2A and Original data 2A). Thus, we chose AN3CA and HEC-1A cell lines to create MGLL overexpression model and knocked-out model respectively. Western blot and qRT-PCR confirmed successful overexpression and knock-down of MGLL (Fig. 2B, C and Original data 2B). Overexpression of MGLL in AN3CA cells promoted the proliferation through MTT, EDU and colony formation assays, while the ability was significantly inhibited upon knock-down of MGLL in HEC-1A cell lines (Fig. 2D–F). Flow cytometry (FCM) assay was used to detect whether the expression of MGLL could affect apoptosis of EAC cells. The results showed that the proportion of apoptotic cells decreased in AN3CA cells with MGLL overexpression and knock-down of MGLL caused significant apoptosis of HEC-1A cells (Fig. 2G). Then we performed Western blot assay to detect the expression of proteins involved in cell cycle and apoptosis. Consistent with above assays, proliferation-related proteins including CyclinD1, CDK4 and anti-apoptotic protein Bcl2 were significantly higher in overexpressing MGLL cells, and pro-apoptotic protein such as cleaved-PARP and cleaved-caspase3 were increased in knock-down MGLL cells (Fig. 2H and Original data 2C). The results suggested that upregulation of MGLL promoted the proliferation and inhibited apoptosis of EAC cells.

### MGLL enhances the invasion and migration of EAC cells

**Fig. 3:**
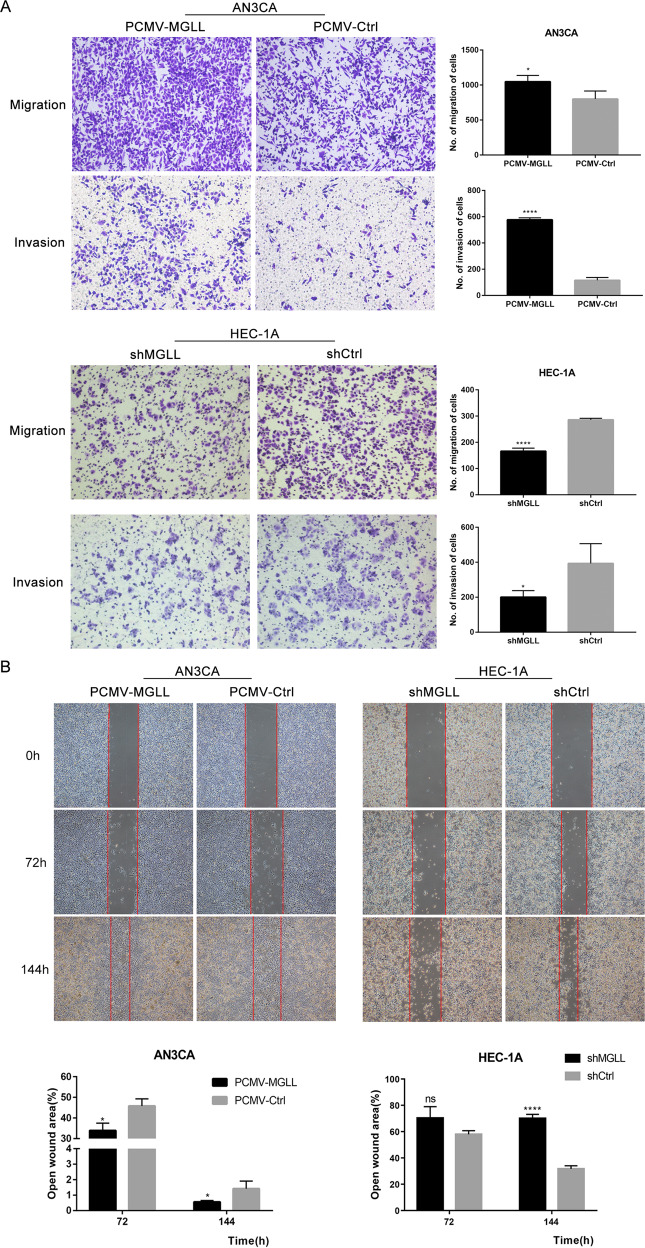

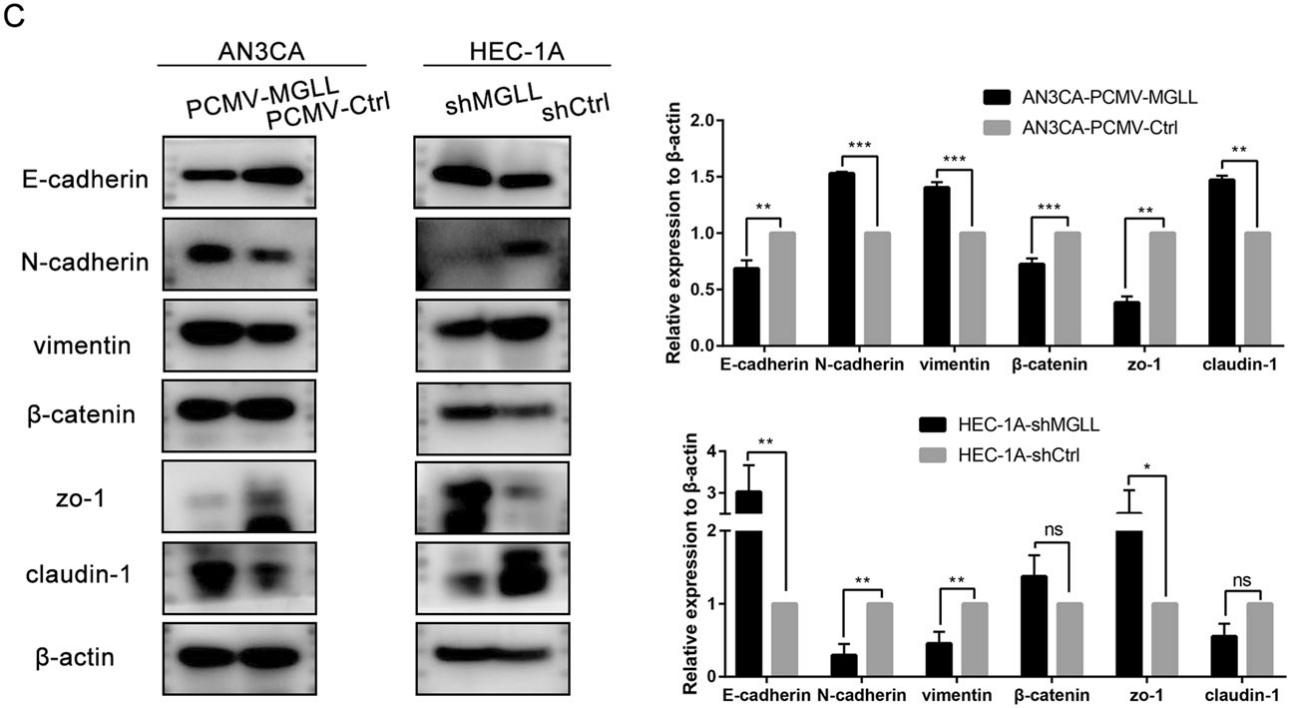
MGLL enhances the invasion and migration of EAC cells. **A** Capacity of migration and invasion after deletion or overexpression of MGLL in EAC cells by transwell assay. **B** Ability of migration after deletion or overexpression of MGLL in EAC cells by wound healing assay. **C** Expression of E-cadherin, N-cadherin, vimentin, β-catenin, zo-1, and claudin-1 were determined by Western blot assay in EAC cells after overexpression or knockdown of MGLL. All experiments were repeated three times at least. *P < 0.05, **P < 0.01, ***P < 0.001 and ****P < 0.0001 for statistical analysis of the indicated groups.

We further investigated whether MGLL affected metastasis of EAC cells. Transwell assay showed the invasion and migration abilities of AN3CA cells in MGLL overexpression group were significantly enhanced compared to the control group, and the abilities of HEC-1A cells in MGLL knock-down group were obviously suppressed (Fig. 3A). As shown in Fig. 3B, compared with AN3CA-PCMV-Ctrl cells, the migration speed of AN3CA-PCMV-MGLL cells was remarkedly faster, and that of HEC-1A-shMGLL cells was significantly slower than that of HEC-1A-shCtrl cells (Fig. 3B). Epithelial mesenchymal transformation pathway (EMT) represents the change of cell metastasis ability, and the decrease of epithelial-related markers such as E-cadherin, β-catenin and ZO-1 indicate the activation of EMT pathway and the enhancement of cell metastasis ability [12]. Therefore, we tested the expression of related markers and found that epithelial-related markers were lower in AN3CA-PCMV-MGLL cell than AN3CA-PCMV-Ctrl cell and higher in HEC-1A-shMGLL than HEC-1A-shCtrl (Fig. 3C and Original data 3A). It indicated that MGLL could promote the migration and invasion of endometrioid adenocarcinoma cells.

### MGLL overexpression counteracts the sensitivity of EAC cells to progesterone

**Fig. 4:**
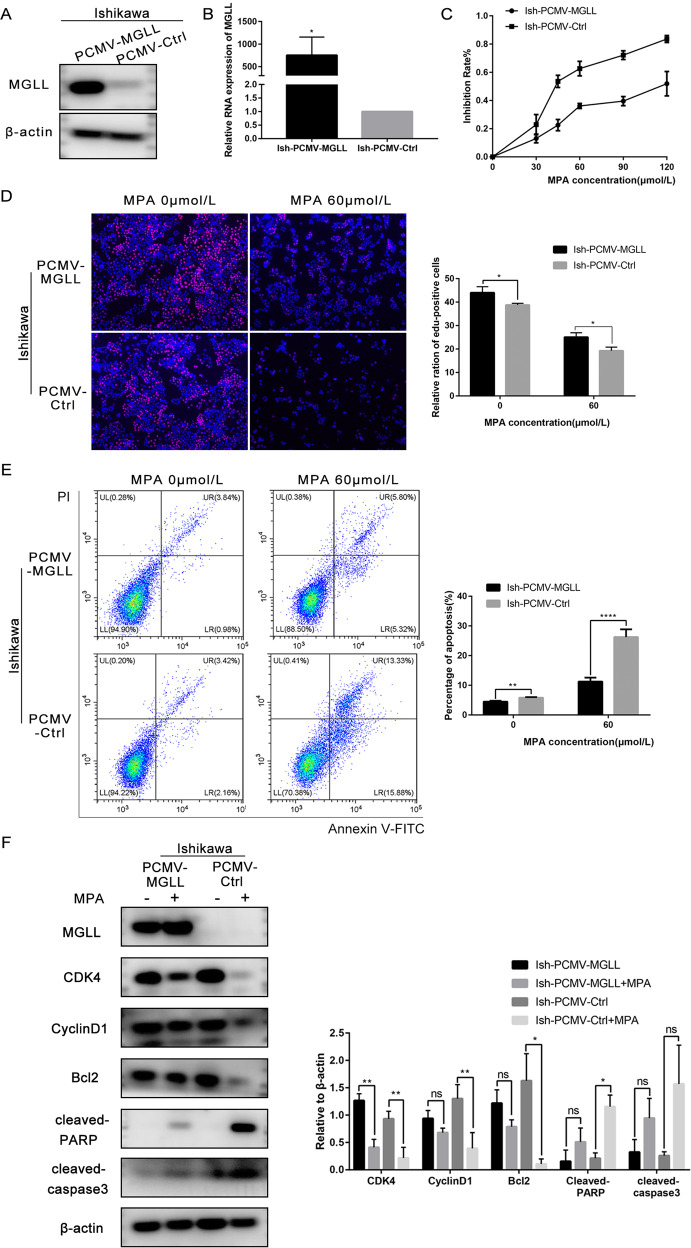

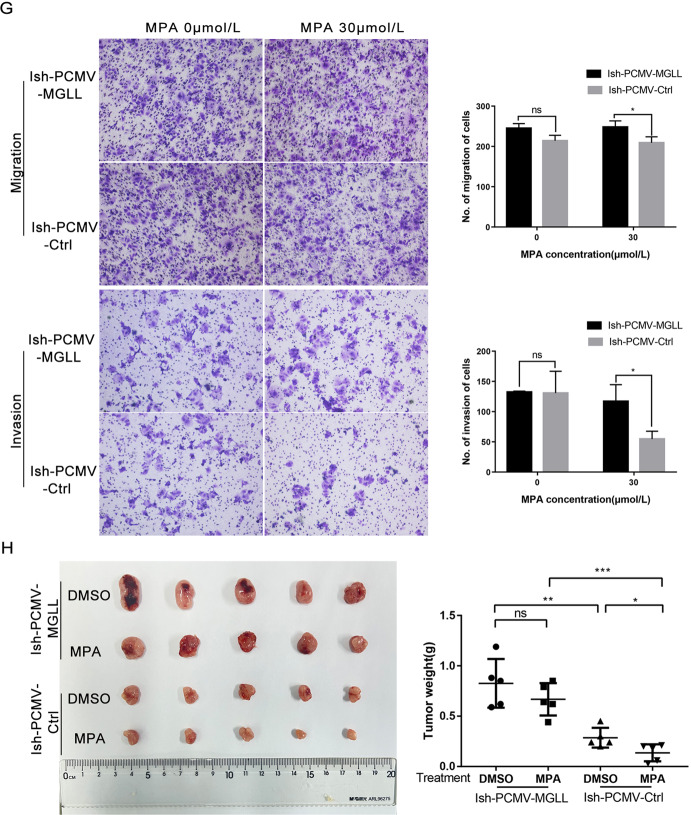
MGLL overexpression counteracts the sensitivity of EAC cells to progesterone. **A** Western blot assay was used to detect the protein expression of MGLL in Ish-PCMV-MGLL and Ish-PCMV-Ctrl cells. **B** qRT-PCR assay was used to detect the mRNA expression of MGLL in Ish-PCMV-MGLL and Ish-PCMV-Ctrl cells. **C** MTT assay was used to detect the viability of Ish-PCMV-MGLL and Ish-PCMV-Ctrl cells with different-dose MPA. **D** The proliferation capacity of Ish-PCMV-MGLL and Ish-PCMV-Ctrl cells with 0 or 60 μM MPA treatment by EDU assay. **E** Apoptotic cells in Ish-PCMV-MGLL and Ish-PCMV-Ctrl groups with 0,60 μM MPA for 48 h respectively. Apoptosis was detected by flow cytometry after staining with FITC Annexin-V and PI. **F** Expression of MGLL, CDK4, CyclinD1, Bcl-2, cleaved-PARP and cleaved-caspase3 were determined by western blot assay in Ish-PCMV-MGLL and Ish-PCMV-Ctrl cells were treated with 0.60 μM MPA for 48 h respectively. **G** Ability of migration and invasion in Ish-PCMV-MGLL and Ish-PCMV-Ctrl cells treated with 0,30 μM MPA for 48 h respectively by transwell assay. **H** The left image shows the tumors transfected with Ish-PCMV-MGLL and Ish-PCMV-Ctrl treated with MPA or DMSO. The right image shows the tumor weights of the four groups of mice. All experiments were repeated three times at least. *P < 0.05, **P < 0.01, ***P < 0.001, and ****P < 0.0001 for statistical analysis of the indicated groups.

We established Ish cells with MGLL overexpression using lentivirus and detected by Western blot and qRT-PCR at the protein and mRNA levels (Fig. 4A, B and Original data 4A). MTT assays showed that the IC_50_ of the Ish-PCMV-MGLL cells almost increased 3-fold compared with Ish-PCMV-Ctrl cells, after 48-hours of treatment with different concentrations of MPA. Ish-PCMV-MGLL cells exhibited a stronger viability at the same concentration of MPA (Fig. 4C). The proliferation ability of Ish-PCMV-MGLL and Ish-PCMV-Ctrl cells treated with MPA at concentrations of 0 and 60 μM for 48 h was detected by EDU assay. The results showed that the proliferation ability of Ish-PCMV-MGLL was increased (Fig. 4D). Furthermore, the percentage of apoptotic cells was lower in the Ish-PCMV-MGLL group than in the Ish-PCMV-Ctrl after MPA treatment by FCM (Fig. 4E). Western blot was then carried out to detect the expression of proteins related to proliferation and apoptosis. Following MPA treatment, when compared with the Ish-PCMV- Ctrl group, the expression levels of CDK4, CyclinD1, and Bcl2, in the Ish-PCMV- MGLL group were significantly up-regulated while the expression of cleaved-PARP was significantly down-regulated (Fig. 4F and Original data 4B). We also detected the migration and invasion of cells in response to MPA through transwell assay, and the ability of Ish-PCMV-MGLL was significantly higher than that of the control group (Fig. 4G). Subsequently, we established nude-mouse xenograft tumor models using Ish-PCMV-MGLL and Ish-PCMV-Ctrl cells. Results showed that MPA treatment showed inconspicuous inhibitory effect on the Ish-PCMV-MGLL tumors. However, in the Ish-PCMV-Ctrl group, MPA treatment significantly inhibit tumor growth (Fig. 4H).

In conclusion, the overexpression of MGLL was related to the occurrence and development of progesterone resistance in EAC.

### MGLL knockdown renders EAC cells more sensitive to progesterone

**Fig. 5:**
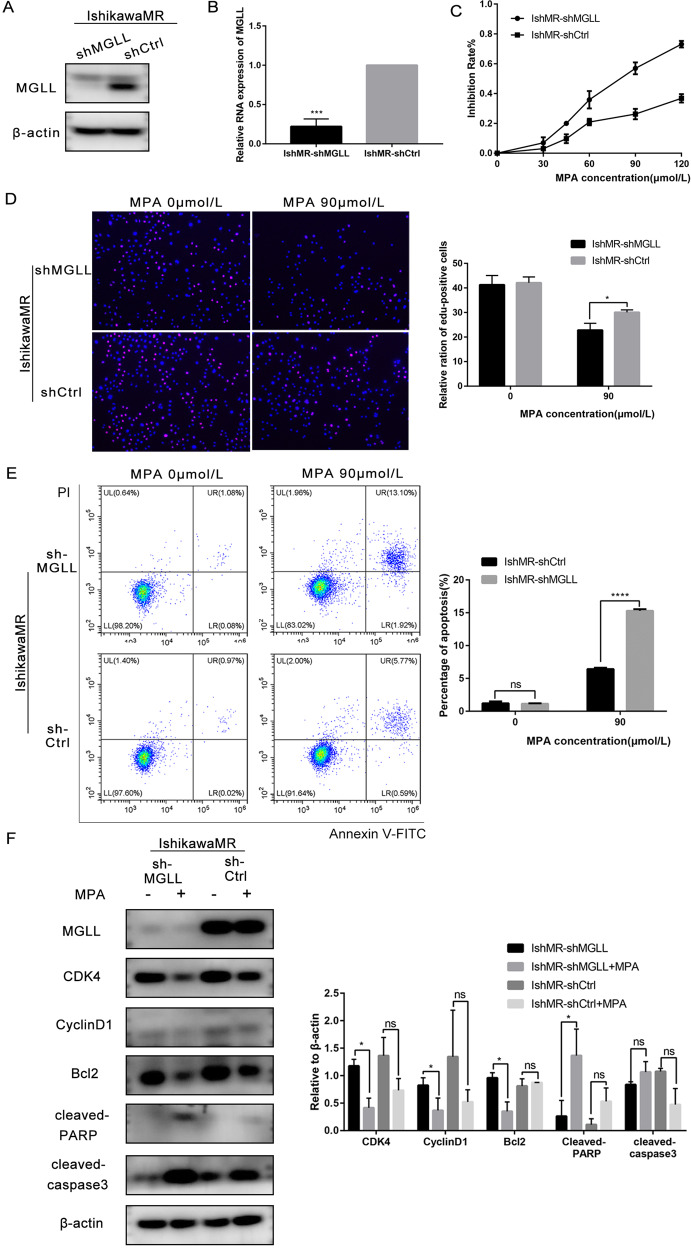

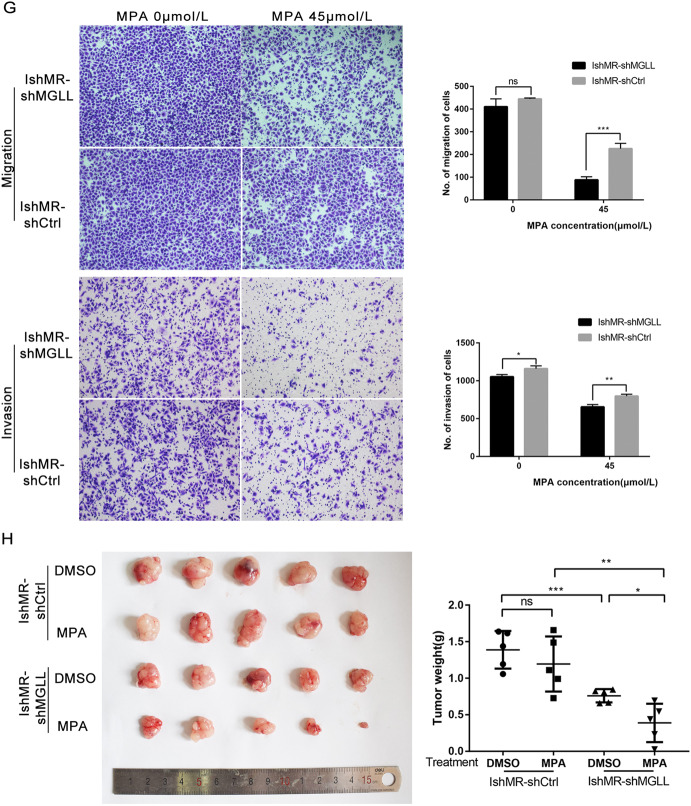
MGLL knockdown renders EAC cells more sensitive to progesterone. **A** Western blot assay was used to detect the protein expression of MGLL in IshMR-shMGLL and IshMR-shCtrl cells. **B** qRT-PCR assay was used to detect the mRNA expression of MGLL in IshMR-shMGLL and IshMR-shCtrl cells. **C** MTT assay was used to detect the viability of IshMR-shMGLL and IshMR-shCtrl cells with different-dose MPA. **D** The proliferation capacity of IshMR-shMGLL and IshMR-shCtrl cells with 0 or 90 μM MPA treatment by EDU assay. **E** Apoptotic cells in IshMR-shMGLL and IshMR-shCtrl groups with 0,90 μM MPA for 48 h respectively. Apoptosis was detected by flow cytometry after staining with FITC Annexin-V and PI. **F** Expression of MGLL, CDK4, CyclinD1, Bcl-2, cleaved-PARP and cleaved caspase3 were determined by Western blot assay in IshMR-shMGLL and IshMR-shCtrl cells were treated with 0,90 μM MPA for 48 h respectively. **G** Ability of migration and invasion in IshMR-shMGLL and IshMR-shCtrl cells treated with 0,45 μM MPA for 48 h respectively by transwell assay. **H** The left image shows the tumors transfected with IshMR-shMGLL and IshMR-shCtrl treated with MPA or DMSO. The right image shows the tumor weights of the four groups of mice. All experiments were repeated three times at least. *P < 0.05, **P < 0.01, ***P < 0.001, and ****P < 0.0001 for statistical analysis of the indicated groups.

We established IshMR-shMGLL cells by lentivirus transfection and investigated expression levels by Western blot and qRT-PCR (Fig. 5A, B and Original data 5A). MTT assays showed that the survival rates of IshMR-shMGLL cells after 48h of treatment with different concentrations of MPA were lower significantly than those of the shCtrl group (Fig. 5C). EDU assays showed that the DNA synthesis of IshMR-shMGLL cells declined sharply when treated with 0, 90 μM of MPA compared with the control group (Fig. 5D). FCM further revealed that the proportion of cells showing signs of apoptosis was significantly higher in IshMR-shMGLL group (Fig. 5E). The detection of proteins related to proliferation and apoptosis further confirmed these experimental findings (Fig. 5F and Original data 5B). Transwell assays were performed to examine the migration and invasion ability of cells in response to MPA, and the capacity of IshMR-shMGLL was inhibited (Fig. 5G). IshMR-shMGLL and IshMR-shCtrl nude-mouse xenograft tumor models were established and then treated with MPA. These experiments revealed that the IshMR-shMGLL group exhibited a greater extent of tumor shrinkage, while the IshMR-shCtrl group only exhibited a slight amount of shrinkage (Fig. 5H).

In summary, the suppression of MGLL in progesterone-resistant cells enhanced the sensitivity of cells to progesterone and reversed progesterone resistance.

### MGLL induces the progesterone resistance in EAC by mediating the generation of ROS and regulating AKR1C1

**Fig. 6:**
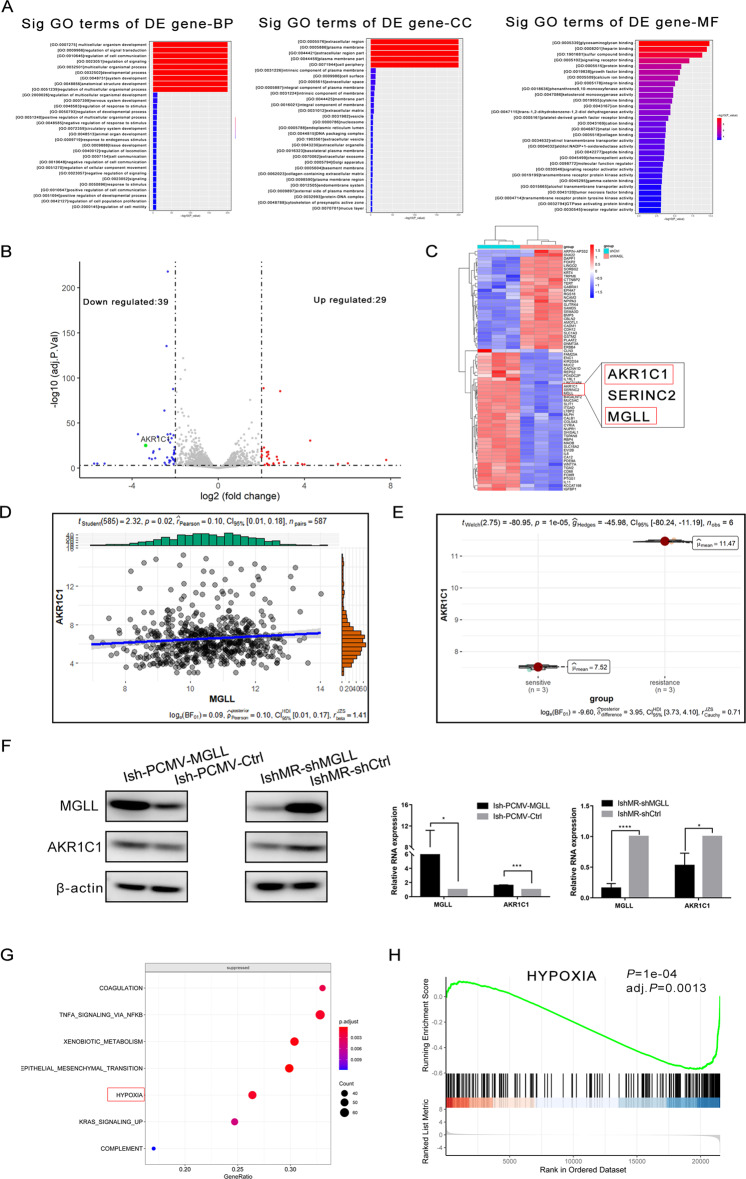

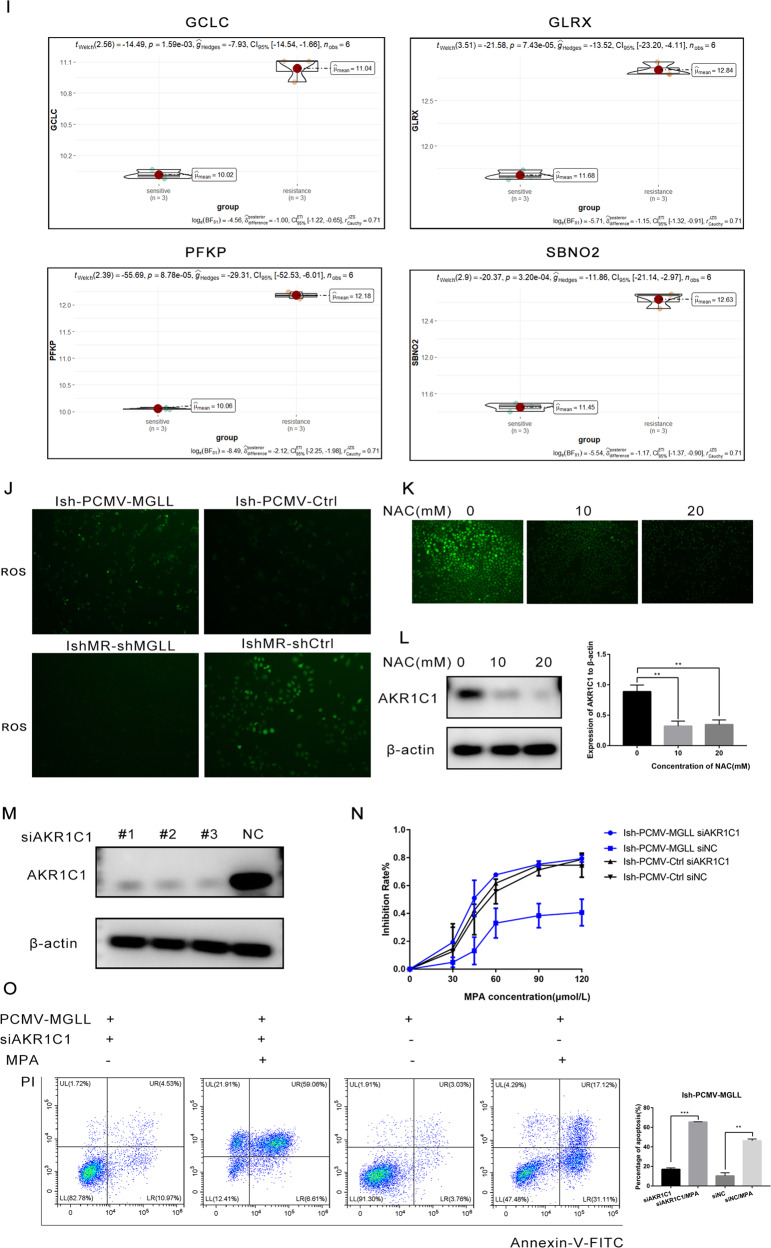
MGLL induces the progesterone resistance in EAC by mediating the generation of ROS and regulating AKR1C1. **A** GO Enrichment of DEGs in IshMR-shMGLL and IshMR-shCtrl cells, including in GO-BP (biological process), GO-CC (cellular component), and GO-MF (molecular function, MF) pathway analysis. **B** Volcano plot of RNA-seq from microarray data in IshMR-shMGLL and IshMR-shCtrl cells. The blue and red dots represent DEGs based on a |FC| of ≥3. The green dot represents AKR1C1. **C** Hierarchically clustered heatmap of differentially expressed genes in IshMR-shMGLL and IshMR-shCtrl cells. AKR1C1 and MGLL are closely located in the heatmap. **D** The relationship of MGLL and AKR1C1 in the TCGA database of EC. **E** The violin plot depicts the expression distribution of AKR1C1 between IshMR and Ish cells. **F** Western blot assay demonstrated that MGLL could regulate the expression of AKR1C1. **G** GSEA analysis revealed that hypoxia was enriched after knockdown of MGLL. **H** GSEA analysis revealed that hypoxia was repressed in MGLL knockdown cells. **I** ROS related markers were highly expressed in the progesterone-sensitive cell line. **J** The generation of intracellular ROS in Ish cells with PCMV-MGLL or PCMV-Ctrl and IshMR cells with shMGLL or shCtrl. The green spots represent the generation of ROS. **K** Levels of ROS decreased with increasing concentrations of NAC in IshMR cells. **L** The expression of AKR1C1 after inhibition of ROS with NAC. **M** Expression of AKR1C1 in Ish-PCMV-MGLL cells after transfected with siAKR1C1 or siNC for 72 by Western blot assay. **N** Growth curves in Ish-PCMV-MGLL cells after transfected with siAKR1C1 or siNC with different-dose MPA by MTT assay. **O** Apoptosis in Ish-PCMV-MGLL cells after transfected with siAKR1C1 or siNC with 30 μM MPA for 48 h by flow cytometry assay. All experiments were repeated three times at least. *P < 0.05, **P < 0.01, ***P < 0.001, and ****P < 0.0001 for statistical analysis of the indicated groups.

To further explore the mechanism underlying the effect of MGLL on biological function and progesterone resistance in EAC, we performed next-generation sequence (NGS) in IshMR-shMGLL and IshMR-shCtrl cells. We did Gene Ontology (GO) enrichment analyses for the altered gene and found that MGLL plays important roles in protein binding, membrane composition and so on (Fig. 6A). Differential genes (DEGs) threshold was set at a adj.P < 0.005 and |fold change(FC)| ≥ 3. A volcano plot was created to show the differentially expressed genes (Fig. 6B). A heat map was also created to show the distribution of differentially expressed genes between two groups (Fig. 6C). We performed qRT-PCR experiments with 4 molecules clustered with MGLL and found that only AKR1C1 expression changed with MGLL expression (Supplement 1A). So, we speculated that MGLL and AKR1C1 might function together . We further confirmed the correlation between MGLL and AKR1C1 using The Cancer Genome Atlas (TCGA) database of EC (Fig. 6D) and found that AKR1C1 was highly expressed in IshMR cell (Fig. 6E). To further confirm the regulation, we examined AKR1C1 expression in Ish-PCMV-MGLL, IshMR-shMGLL and corresponding control cell lines respectively, and found that expression of AKR1C1 decreased in IshMR-shMGLL cell lines and increased in Ish-PCMV-MGLL cell lines compared to control groups. It indicated that MGLL could affect AKR1C1 (Fig. 6F and Original data 6A). GSEA analysis indicated that MGLL was able to lead to alterations of hypoxic microenvironment within tumor cells (Fig. 6G, H). The NGS results showed that ROS related markers (GCLC, GLRX, PFKP and SBNO2) were highly expressed in the progesterone-sensitive cell line (Fig. 6I). Subsequently we found that MGLL led to an increase in ROS generation (Fig. 6J). It has been documented that MGLL could induce an increasing level of ROS. After determining that NAC inhibited ROS generation, AKR1C1 expression was also decreased (Fig. 6K, L and Original data 6B). The above results showed that MGLL may induce the development of progesterone resistance in EAC by increasing intracellular ROS and further activating AKR1C1 expression.

To validate the above conclusions, we knocked down AKR1C1 in Ish-PCMV-MGLL cells by siRNA and then carried out rescue experiments to detect the sensitivity to MPA. The knockdown efficiency of AKR1C1 was verified by Western blot assay (Fig. 6M and Original data 6C). The results of MTT assay and apoptosis assay showed that interfering with AKR1C1 in Ish-PCMV-MGLL cells restored the sensitivity to MPA (Fig. 6N, O).

To draw a conclusion, MGLL is involved in progesterone resistance in EAC by increasing intracellular ROS and activating AKR1C1 expression.

### ABX-1431 inhibited the growth of EAC and reversed progesterone resistance in vitro by inhibiting the expression of MGLL

**Fig. 7:**
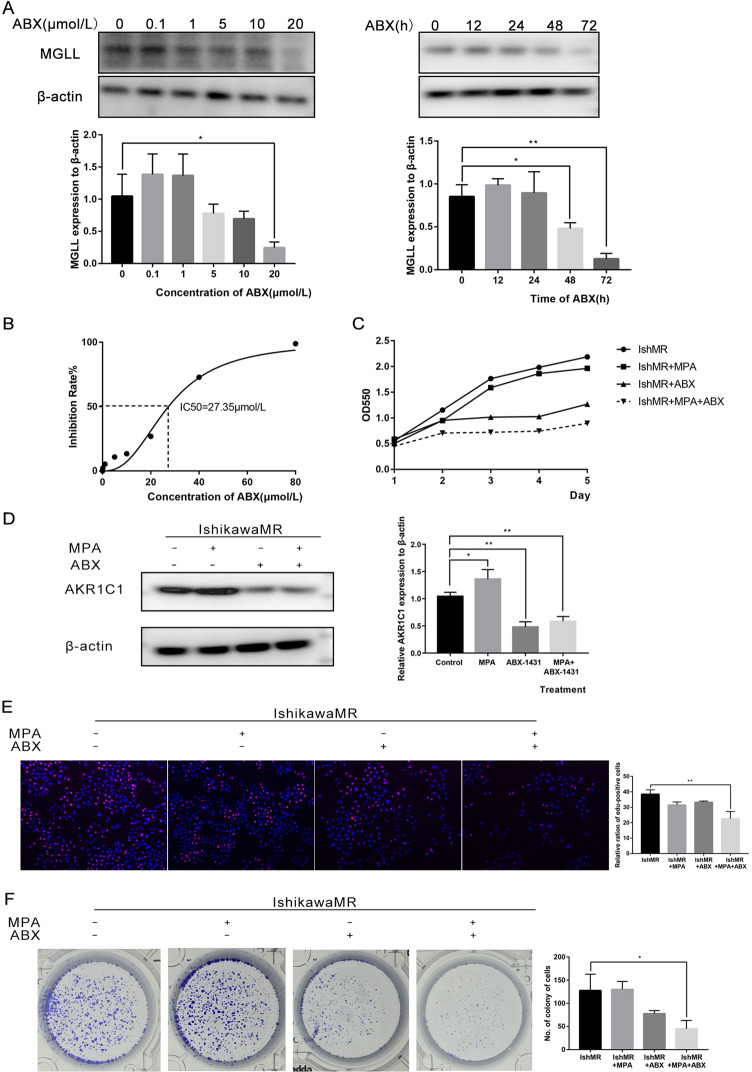

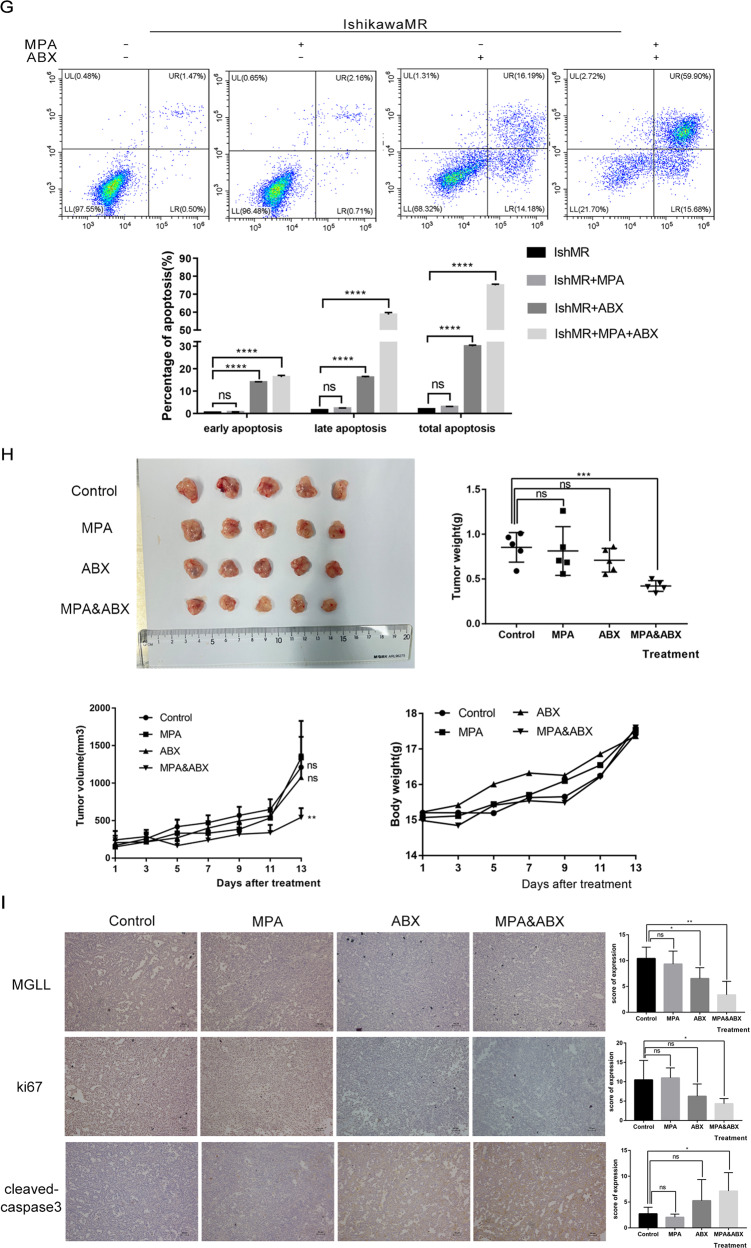
ABX-1431 enhances the sensitivity of EAC to progesterone by inhibiting MGLL in vivo and vitro. **A** Expression of MGLL in IshMR cells treated with different concentration and time of ABX-1431. **B** MTT assay showed the inhibition rate of IshMR cells treated with different concentrations of ABX-1431 and the IC50 of ABX-1431 on IshMR cells. **C** The effects of MPA and ABX-1431 treatment on cell viability by the CCK8 assay. **D** Western blot assay showed simultaneous application of ABX-1431 and MPA could inhibit AKR1C1 expression. **E** The proliferation rate of IshMR treated with MPA or/and ABX-1431 by EDU assay. **F** The colony-forming ability of IshMR treated with MPA or/and ABX-1431. **G** The apoptosis of IshMR cells treated with MPA or/and ABX-1431 by flow cytometry. **H** Images of tumors of IshMR cells with MPA, ABX-1431 and combined drug. And the volume and weight of tumor and the body weight of the four groups of mice. **I** Representative images of IHC staining of MGLL, cleaved-caspase3 and Ki67 in tumor tissues. Scale bar: 10 μm. All experiments were repeated three times at least. *P < 0.05, **P < 0.01, ***P < 0.001, and ****P < 0.001 for statistical analysis of the indicated groups.

Given that MGLL plays an important role in progesterone resistance, it is bold to hypothesize that targeted inhibition of MGLL by ABX-1431 may have significant therapeutic advantages. As shown in Fig. 7A (Original data 7A), ABX-1431 could inhibit the expression of MGLL, and the inhibitory effect became stronger with the increase of concentration and extension of time. We determined that the IC50 of ABX-1431 on IshMR cells was 27.35 μM by MTT assay (Fig. 7B). Then we tested the effect of ABX-1431 and MPA on the viability of IshMR cells through CCK8, and found that 30 μM MPA alone had no significant inhibitory effect on the viability of IshMR cells, while 20 μM ABX-1431 could significantly inhibit the viability of EAC cells. Moreover, treatment with ABX-1431 combined with MPA further reduced cell viability (Fig. 7C). Meanwhile, the results showed that simultaneous application of ABX-1431 and MPA could inhibit AKR1C1 expression (Fig. 7D and Original data 7B). Then, EDU was applied to detect the ratio of proliferating cells and showed that combination of treatment remarkedly inhibited proliferation (Fig. 7E). The long-term effects of two drugs on the proliferation ability of progestin-resistant cells were determined by colony formation assay, and the results were consistent with MTT (Fig. 7F). Apoptosis assay demonstrated that the combination of ABX-1431 and MPA for 48h, significantly promoted apoptosis compared with the control, MPA and ABX-1431 groups (Fig. 7G). Together, these data suggested that ABX-1431 increased the sensitivity of EAC cells to progesterone.

### Co-treatment with ABX-1431 and MPA synergistically inhibited the proliferation of progesterone-resistant EAC in vivo

In order to further verify the sensitizing effect of ABX-1431 on progesterone, IshMR cell xenograft models were established and treated with DMSO, MPA, and/or ABX-1431, respectively. It was found that the combination of MPA and ABX-1431 significantly inhibited the growth of tumors compared with the control group and single drug group, and there was no significant difference in the body weight of mice among the four groups (Fig. 7H). Consequently, we detected markers associated with cell proliferation and apoptosis through IHC assay and validated the same results (Fig. 7I). It can be seen that in vivo, ABX-1431 was able to enhance the sensitivity of EAC to progesterone and inhibit the growth of tumors.

## Discussion

In recent years, with the increasing incidence of endometrial adenocarcinoma (EAC) and the younger patients, more and more population tend to choose conservative treatment to preserve reproductive function. However, progesterone resistance is a difficult problem in the treatment of EAC. Many theories are related to progesterone resistance, such as PGR-ER imbalance theory and abnormal activation of multiple signaling pathway including PI3K-AKT, Nrf2-survivin and autophagy pathway [13–15], but the specific molecular mechanism of progesterone resistance is still unclear. When progesterone resistance occurs in tumors, progestin can’t exert its anticancer effects, but shows the function of promoting cancer cell proliferation and metastasis. Therefore, it is urgent to explore the molecular mechanism related to progestogen resistance in order to reverse progestogen resistance, improve the prognosis of patients and preserve the reproductive function of young patients.

In consideration of the problem, our group previously established progesterone resistance cell line based on Ishikawa cell and performed NGS in progesterone sensitivity cell (Ishikawa) and progesterone resistance cell (IshMR) [16]. In this study, we firstly analyzed the RNA-seq results of Ishikawa cell and IshMR cell and screened out that MGLL expression was significantly increased in progesterone resistant cell lines [17]. MGLL is a key hub in the lipid signaling network and has been found to be involved in the development of varieties of tumor, such as breast cancer and melanoma cancer [18, 19]. Subsequently, we confirmed that the expression of MGLL was elevated in EAC through the analysis of paired sample data of EAC in GEO database and the detection of tumors and adjacent tissues of EAC patients in Qilu Hospital of Shandong University. Meanwhile, we further verified that MGLL expression was obviously increased in progesterone resistance cell line and samples. Therefore, we hypothesized that MGLL was highly expressed in EAC and has a correlation with progesterone resistance.

Subsequently, we overexpressed and knocked down MGLL in EAC cell lines, respectively and conducted functional experiments in vivo and in vitro.We verified that MGLL could promote the proliferation and inhibit apoptosis of tumor cells. It has been confirmed in the literature that the expression of MGLL in the primary lesion is higher in deeper areas of the tumor, indicating that tumor cells overexpressing MGLL are more aggressive [19]. So we also focused on the metastatic ability of cells with different expression levels of MGLL and verified it. Moreover, several studies have shown that MGLL is able to act by regulating the EMT pathway [18, 20], so we verified that MGLL promoted the metastasis of EAC cells by regulating the EMT pathway.

In order to determine whether MGLL plays an important role in progesterone resistance of EAC, we conducted functional studies by overexpressing and knocking down of MGLL in EAC progesterone-sensitive cell lines and progesterone-resistant cell lines, respectively. The results showed that MGLL significantly improved the viability of Ish cells treated with MPA and induced progesterone resistance. In contrast, knocked down MGLL inhibited the proliferative capacity of IshMR cells, making them sensitive to progesterone. The results in vivo were consistent with those in vitro. These results indicated that the different expression levels of MGLL in tumors could affect the sensitivity of EAC cells to progesterone. Therefore, we believed that MGLL can promote the progress of EAC and participate in the development of progesterone resistance in EAC.

Then, we performed NGS in IshMR-shMGLL cells and control cells and found that AKR1C1 expression was significantly reduced in IshMR-shMGLL cells. The location of two genes in the heatmap suggested that MGLL and AKR1C1 might function together, then we further verified that AKR1C1 could be affected by MGLL expression. GSEA enrichment analysis showed that overexpression of MGLL resulted in the development of hypoxia in the tumor microenvironment. At present, it is generally believed that the contradiction between the rapid growth of tumor tissue and the incomplete vascular system in tumor tissue leads to insufficient oxygen supply in tumor tissue, presenting a hypoxia microenvironment [21]. Tumor cells metabolize energy through anaerobic glycolysis, resulting in the accumulation of lactic acid and increased production of reactive oxygen species. Tumor cells in hypoxia microenvironment can escape drugs targeted at cell division by producing ROS and lead the resistance [22, 23].So we further examined the effect of MGLL on ROS generation and found that MGLL overexpression could lead to increased generation of ROS in EAC cells. Several literatures have demonstrated that ROS can induce the expression of AKR1C1 [24–26], and our experiments obtained the same results in EAC. The aldosterone reductase superfamily(AKRs) is a nicotinamide adenine dinucleotide phosphate (NADPH) -dependent oxidoreductase, and previous studies have shown that AKR1C1 can degrade progesterone into metabolite 20α-DHP, which binds to the specific plasma membrane and affects mitosis and cytoskeletal formation [27, 28]. Overexpression of AKR1C1 may lead to inhibiting the production of progesterone receptors (PGR) and affect progesterone action [29]. Therefore, we speculated that MGLL might regulate the generation of ROS in EAC cells together with promoting the expression of AKR1C1 and accelerate the degradation of progesterone, leading to the development of progesterone resistance. To test the hypothesis, we interfered with AKR1C1 expression in cells overexpressing MGLL and found that no progesterone-resistant effect occurred in the cells. The above results suggested that MGLL is involved in progestogen resistance in EAC by activating AKR1C1. Because there are many factors affecting ROS in cells, we need further experiments to verify the direct factors of ROS generation induced by MGLL and further explore whether AKR1C1 is affected by ROS or AKR1C1 acts as a sensor of ROS changes.

Finally, in order to explore efficient strategies to reserve progesterone resistance in EAC, we selected MGLL inhibitor for experiments according to previous researches. ABX-1431 is a lead compound for clinical evaluation based on optimized activity and selectivity for multiple human protein tissues [9]. Currently, ABX-1431 has successfully completed phase I clinical trials showing that the compound is well tolerated and safe, and phase II clinical studies of ABX-1431 are ongoing [10]. Therefore, we selected ABX-1431 as a MGLL inhibitor to verify whether it could inhibit the proliferation of EAC and reverse progesterone resistance. MTT, EDU, clony formation and apoptosis assays proved that the application of ABX-1431 could not only inhibit the proliferation and promote apoptosis of EAC cells, but also sensitize the effect of progesterone. Therefore, we believed that the combination of ABX-1431 and progesterone could be used in clinical treatment to improve the sensitivity and efficacy of EAC patients treated conservatively with progesterone.

In summary, it is the first study to confirm that MGLL is one of the key molecules involved in the development of EAC and progesterone resistance. Based on our research, it may act mainly by affecting the hypoxia microenvironment in tumor cells and regulating the expression of AKR1C1. We hypothesize that the high expression level of MGLL could be considered as a standard biomarker to evaluate the efficacy of progesterone in EAC patients. Our data also demonstrate that application of ABX-1431, an inhibitor of MGLL, can reverse progesterone resistance in patients with EAC and can be expected to achieve targeted therapy. Combination of ABX-1431 and progesterone can reserve progesterone resistance effectively and may provide new therapeutic strategies for clinical practice.

## Materials and methods

### Cell lines and cell culture

Ishikawa cells (referred to throughout this paper as ‘Ish’), AN3CA, RL-95-2, HEC-1A, and KLE cells were purchased from Shanghai Zhong Qiao Xin Zhou Biotechnology Co. Progesterone resistant cells which we referred to as IshikawaMR (or ‘IshMR’) were previously obtained by our group via the increasing MPA concentration gradient method [30]. Ish and IshMR cells were cultured in RPMI1640 medium (BI, USA) containing 10% fetal bovine serum (BI, USA), RL-95-2 and HEC-1A cells were routinely grown in M5A media, AN3CA cells were cultured in RPMI-DMEM medium (BI, USA), and all cell lines were cultured at 37 °C in a 5% CO_2_ humidified atmosphere. 10 μM MPA was added to the medium containing the IshMR cells to maintain resistance.

### Western blot assay

Cells were collected, lysed using a mixture containing RIPA, PMSF, and NaF, and the supernatant was taken after centrifugation and sonication to determine the protein concentration using the BCA method (Tiangen Biotech Co., Ltd., Beijing, China). Protein was separated by SDS-PAGE and transferred to PVDF membranes (Millipore, Bedford, MA, USA). The band was cropped according to the weight of the target gene and placed in the antibody overnight, and placed in the secondary antibody for 2 h at room temperature. Protein bands were detected by ImageQuant LAS4000 (General Electric Company, Boston, MA, USA) and quantified by ImageJ software. β-actin was detected as a loading control.

### Quantitative real‑time transcription‑polymerase chain reaction(qRT-PCR)

Total RNA was extracted from cells or tissues and the concentration and purity was evaluated with a spectrophotometer (Thermo Fisher Scientific Inc., MA, USA). RNA was then reverse transcribed into cDNA (3000 ng/10 μl reaction system). PCR reactions were then performed on a StepOne ™ PCR amplifier (Applied Biosystems, USA) with SYBR-green (TAKARA, Japan) in a 10 μl reaction system; β-actin was used as a control. The primers used are shown in the Supplementary information (Supplement Table 1).

### Tissue samples and immunohistochemistry assay

The EAC and adjacent tissues for Western Blot, RT-qPCR and IHC were from patients with primary EAC without previous therapy from Qilu Hospital of Shandong University. We acquired tissues from 37 patients who underwent progesterone treatment at Qilu Hospital of Shandong University between 2010 and 2020. The tissues were collected from the Pathology Department at Qilu Hospital. These patients did not have any other diseases of the reproductive system. The pathological diagnosis of endometrial carcinoma or hyperplasia was made in accordance with the latest National Comprehensive Cancer Network (NCCN) guidelines. All patients received medroxyprogesterone acetate for at least 6 months and were followed up regularly. Complete response (CR) was defined as the absence of residual hyperplasia or cancer in more than 95% of the tissue. Partial response (PR) was defined as <50% of residual hyperplastic glands. If more than 50% of residual hyperplasia was evident, and the extent of hyperplasia was similar to or worse than before progesterone treatment, then the patients were classified as no change(NC) or progressive disease (PD) [31–33].

All human tissue samples were dehydrated for 1 h and dewaxed with xylene and ethyl alcohol. We then used a microwave antigen retrieval technique to repair antigen. We then stained the tissues antibodies against MGLL (1:300), ki67(1:500) and cleaved-casepase3(1:800). Positive staining was subsequently visualized with 3,3’-Diaminobenzidine (DAB) and counterstained with hematoxylin Detailed experimental and analytical methods for IHC were described previously [34].

### Antibodies and agents

Antibodies used in WB and IHC experiments were following: MGLL (Abcam, ab234701), CDK4 (Cell Signaling Technologies, #12790), Cyclin D1 (Cell Signaling Technologies, #2978), cleaved-PARP (Cell Signaling Technologies, #5625), Bcl2 (Cell Signaling Technologies, #4223), cleaved-casepase3 (Cell Signaling Technologies, #9664), EMT kit(Cell Signaling Technologies, #9782), β-actin (Cell Signaling Technologies, #4970), Ki67 (Abcam, ab92742),AKR1C1(Abcam,ab179448), mouse IgG (Cell Signaling Technologies, #7076), and rabbit IgG (Cell Signaling Technologies, #7074). Medroxyprogesterone acetate(MPA) and ABX-1431 were obtained from Abcam and Selleck, respectively, and were both diluted in DMSO.

### CCK8 assay

0.3 × 10^4^ cells were seeded into 96-well plates, and 10 μlCCK8 was added to each well after cell attachment, and OD550 absorbance was measured 1 h later, as the first day, and then at the same time every day.

### MTT assay

MTT assays were used to analyze cell viability and determine the 50% inhibitory concentration (IC_50_); 0.3 × 10^4^ cells were seeded into a 96-well plate, and different concentrations of MPA were added to it the next day. After culture for 48 h, 10 μl MTT (5 mg/mL in PBS) solution was added to each well, as if it had been incubated in an incubator for 4 h. Formazan crystals were dissolved in 150 μl of dimethylsulfoxide (DMSO; Sigma-Aldrich, St Louis, MO, USA). The absorbance at OD550 was measured, and the inhibition rate of the drug on the cells was calculated.

### Colony formation assay

600 cells were seeded into six-well plates, attached or treated with MPA, and cultured for about 5–14 days when the cell density was appropriate, the cells were fixed with methanol and stained with crystal violet (Beyotime, Beijing, China). Photographs were taken for statistics.

### EDU incorporation assay

0.6 × 10^4^ cells were seeded into 96-well plates, and after adherent or addition of MPA treatment, cells were fixed and stained for proliferating cells using the EDU kit, and finally Hoechst -labeled cells were used for statistics.

### Evaluating cellular apoptosis by flow cytometry

After cells were attached or treated with MPA for 48 h, cells were collected for apoptosis. Then, we performed a cell apoptosis assay using a FACS flow cytometer and a FITC Annexin V Apoptosis Detection Kit (BD Bioscience Pharmingen, San Diego, CA, USA); the kit was used in accordance with the manufacturer’s instructions. Finally, data were analyzed by Cell Quest software (Becton Dickinson, Franklin Lakes, NJ, USA).

### Transwell assay

Transwell assays were performed in transwell inserts (8-μm pore size, BD Biosciences, USA) inserted into 24-well plates without or with Matrigel (BD Biosciences, USA). The upper chamber was coated with 200 μl of serum-free medium containing 8 × 10^4^ cells (for migration) or 12 × 10^4^(for invasion), while the lower chamber contained 700 μl of medium supplemented with 20% FBS. After incubation at 37 °C for the appropriate time, cells that had migrated to the lower surface of the membrane were fixed with methanol, stained with 0.5% crystal violet, and observed and quantified under a light microscope.

### Wound healing assay

20 × 10^4^ cells were seeded into 24-well plates, and when the cell density reached 90%, the wound was scratched using a 10 μl pipette tips, and the cells were cultured until they reached confluence and photographed at 0, 72, 144 h.

### Xenograft model

Four-week-old female BALB/c mice were injected with 1 × 10^7^ cells cells into the right armpit. When the tumor diameter was about 5 mm, they were randomly divided into 4 groups and treated with intraperitoneal injection of drugs according to the experimental design. For MPA, the dose was 100 mg/kg/ bodyweight; for ABX-1431, the dose was 1 mg/kg, and the control group received the same amount of DMSO. Mouse body weight and tumor size were measured every two days. Twenty mice were treated with drugs, euthanized, and the tumors were removed. Tumor size = width^2^ × length/2. The animal experiments in our study were approved by the Ethics Committee of Shandong University.

### Next-generation sequence

The high-throughput mRNA-Seq experiments were conducted by Sequencing company. Briefly, IshMR cells were transfected with shMGLL or Ctrl(*n* = 3) and total RNAs were extracted using TRIzol reagent. Genes with an adj.P < 0.005 and |fold change(FC) | ≥3 found by DESeq were set as differentially expressed genes (DEGs). Gene function was annotated based on Gene Ontology (GO)database and The database of Kyoto Encyclopedia of Genes and Genomes (KEGG) database. The Gene Set Enrichment Analysis (GESA) enrichment analysis was implemented by the clusterProfiler R package.

### Measure of ROS and inhibitor

Intracellular hydrogen peroxide levels were measured using 2,7-dichlorodihydrofluorescein diacetate (DCFH-DA; Beyotime Biotechnology, China). The cultured cells were washed once and incubated with DCFH-DA (20 μM, 30 min). DHE fluorescence was detected with a fluorescence microscope. Experiments were performed using N-acetylcysteine (NAC) (Selleck Chemicals, Houston, TX, United States) as an ROS inhibitor.

### Statistical analysis

Data were analyzed using GraphPad Version 7.0 software.Statistical significance was determined by Student’s *t* tests, one-way analysis of variance (ANOVA) and two-way ANOVA. All experiments were repeated at least three times. Statistical significance was set at *P* < 0.05.

## Supplementary information

Reproducibility Checklist

Supplement 1

Original data 1

Original data 2

Original data 3

Original data 4

Original data 5

Original data 6

Original data 7

Author Contribution Statement

Supplement Table 1

## Data Availability

The datasets used and/or analyzed during the current study are available from the corresponding author on reasonable request.
