# Supplementary figures and images for: ABX-1431 inhibits the development of endometrial adenocarcinoma and reverses progesterone resistance by targeting MGLL
Source: Cell Death Dis. 2022 Dec 23;13(12):1067. doi: 10.1038/s41419-022-05507-z (PMC9780207; doi:10.1038/s41419-022-05507-z)

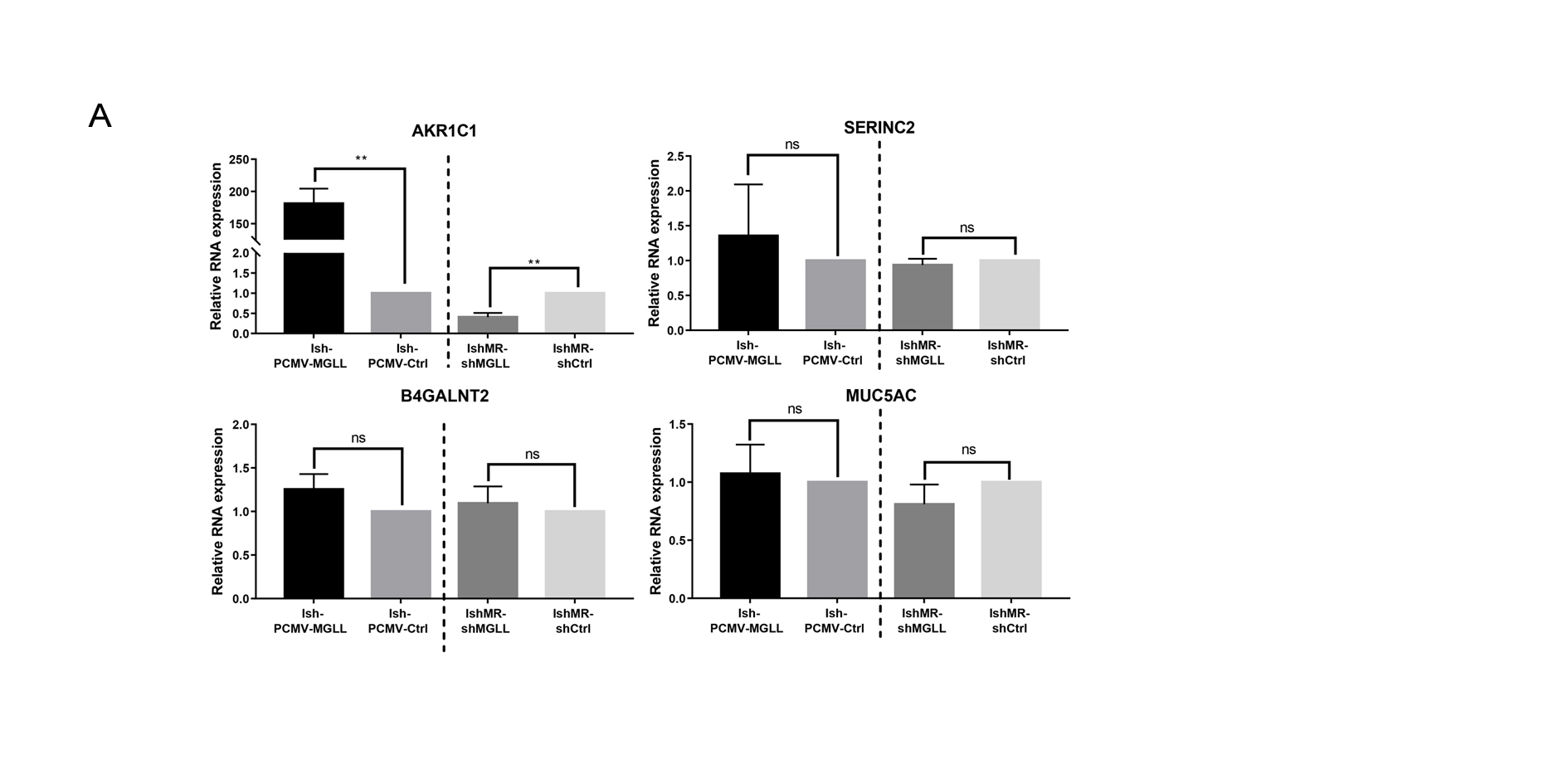

Supplement: Supplementary file 2 — Supplement 1 [file 41419_2022_5507_MOESM2_ESM.jpg]

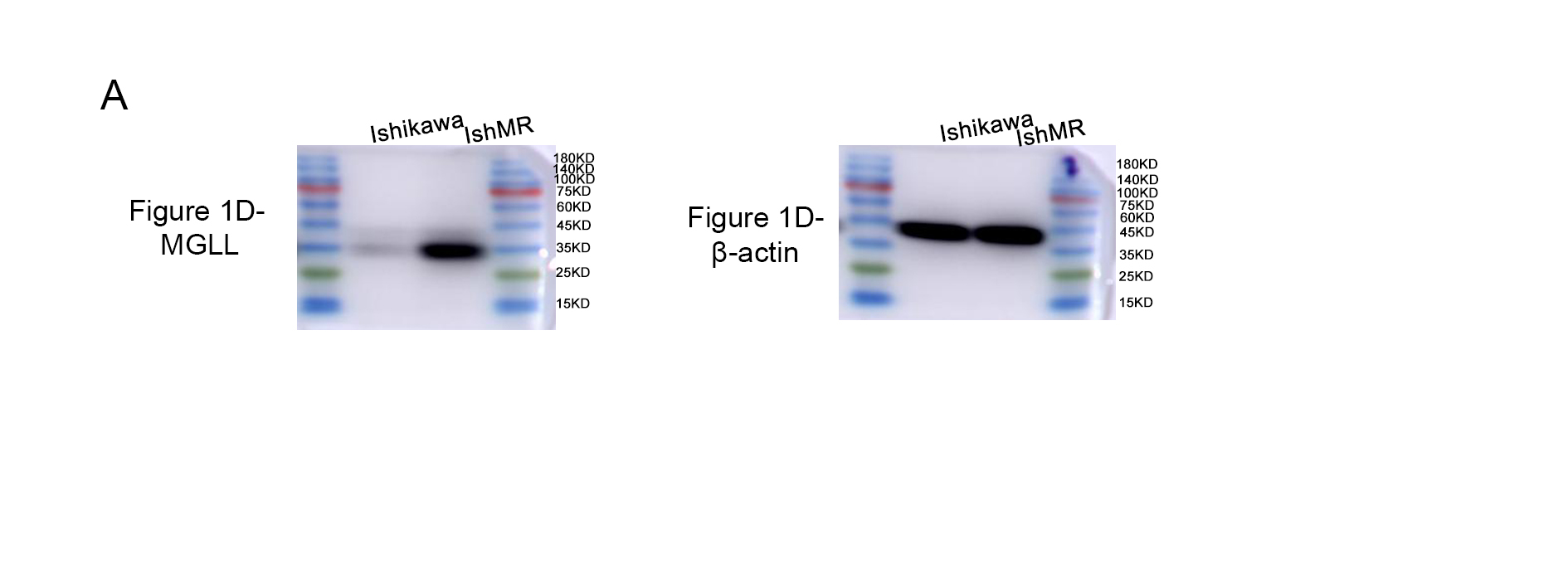

Supplement: Supplementary file 3 — Original data 1 [file 41419_2022_5507_MOESM3_ESM.jpg]

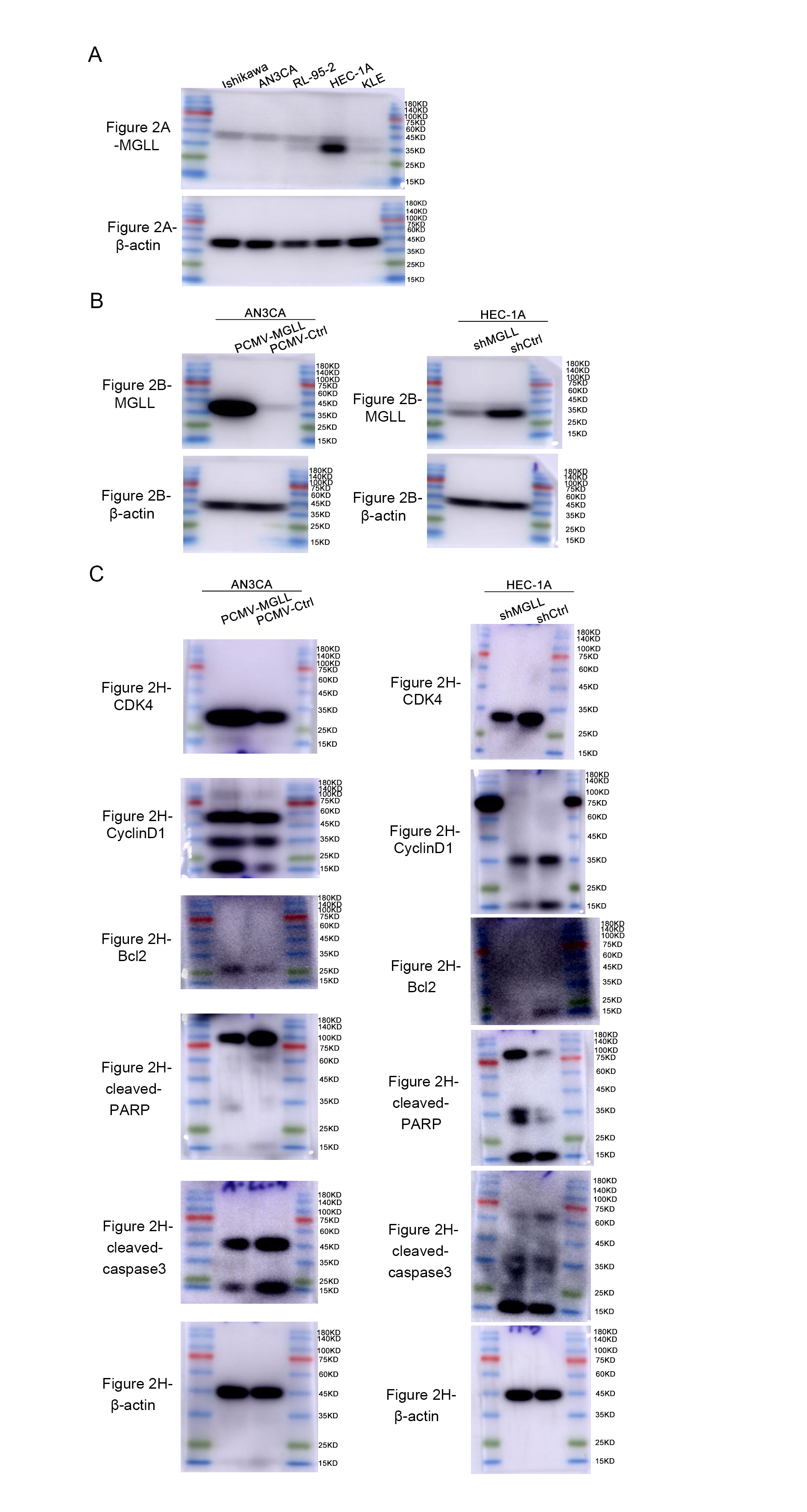

Supplement: Supplementary file 4 — Original data 2 [file 41419_2022_5507_MOESM4_ESM.jpg]

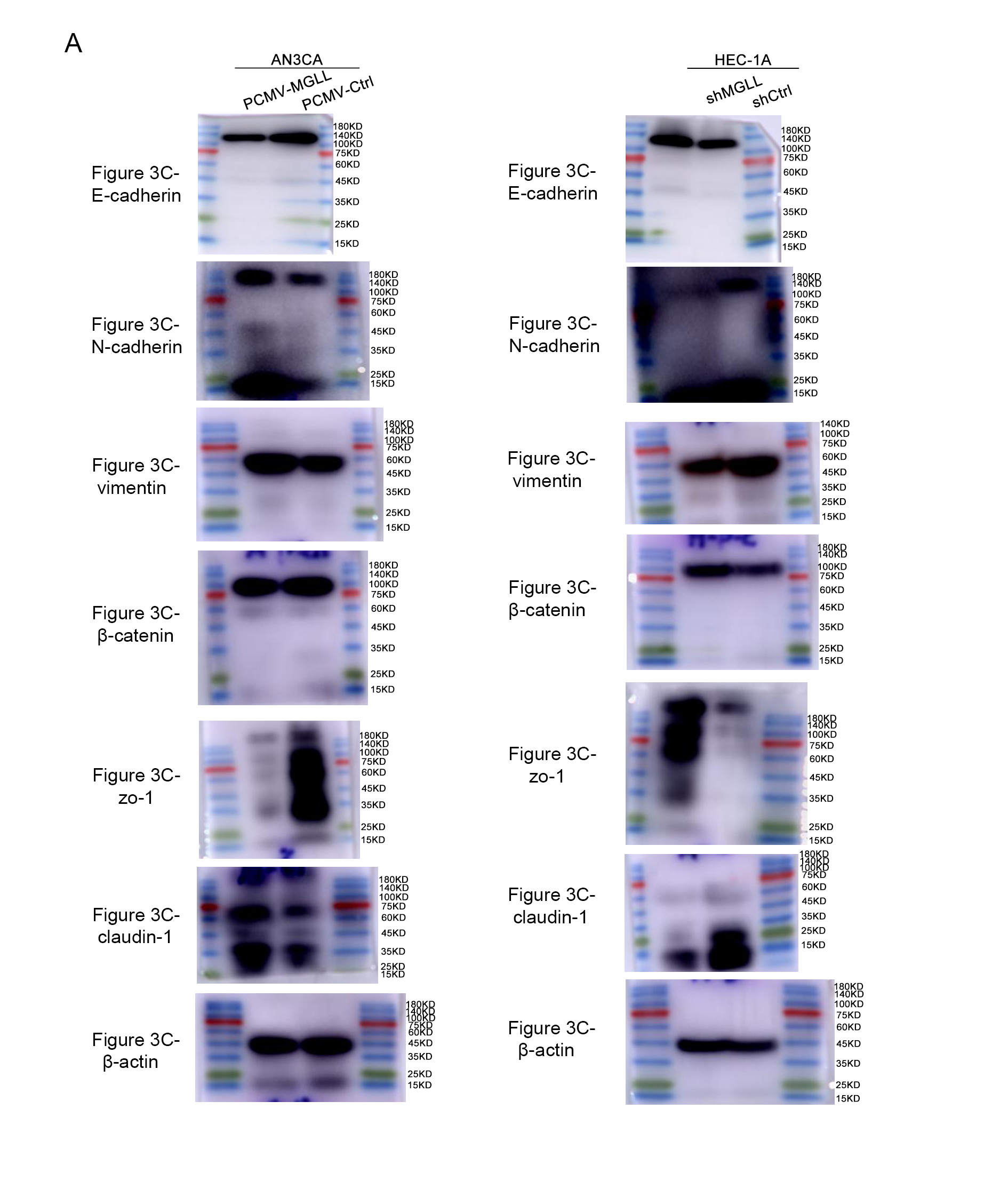

Supplement: Supplementary file 5 — Original data 3 [file 41419_2022_5507_MOESM5_ESM.jpg]

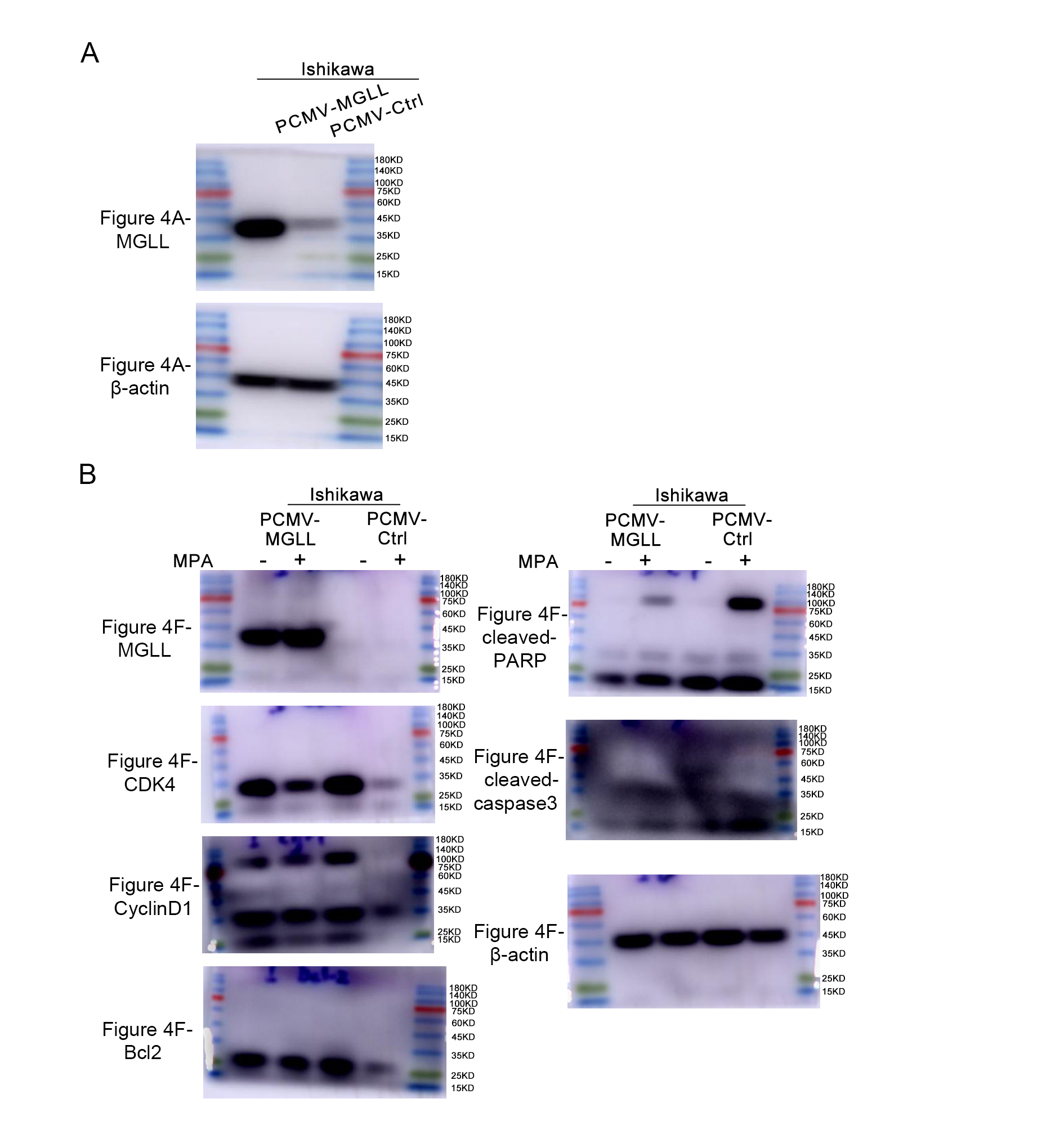

Supplement: Supplementary file 6 — Original data 4 [file 41419_2022_5507_MOESM6_ESM.jpg]

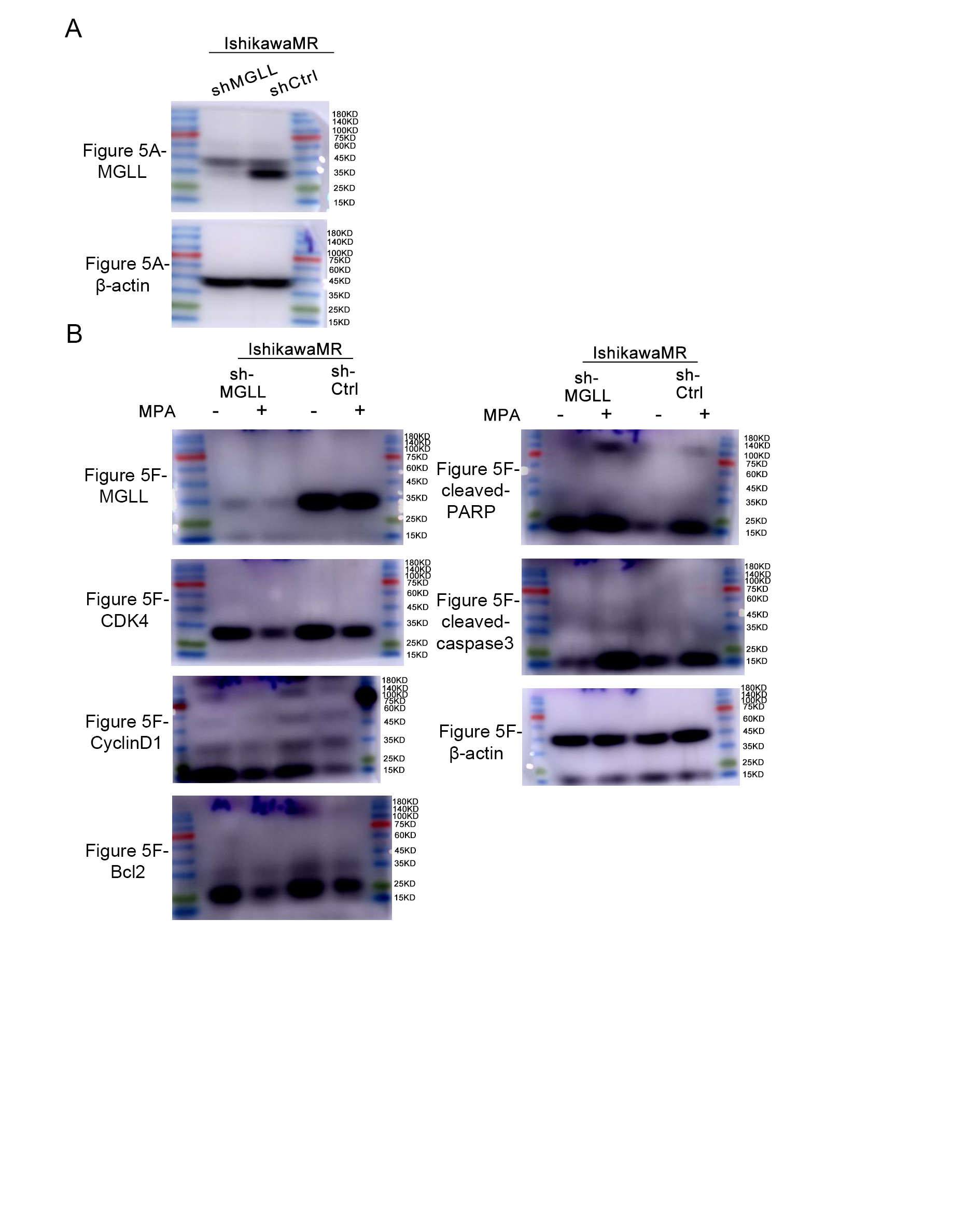

Supplement: Supplementary file 7 — Original data 5 [file 41419_2022_5507_MOESM7_ESM.jpg]

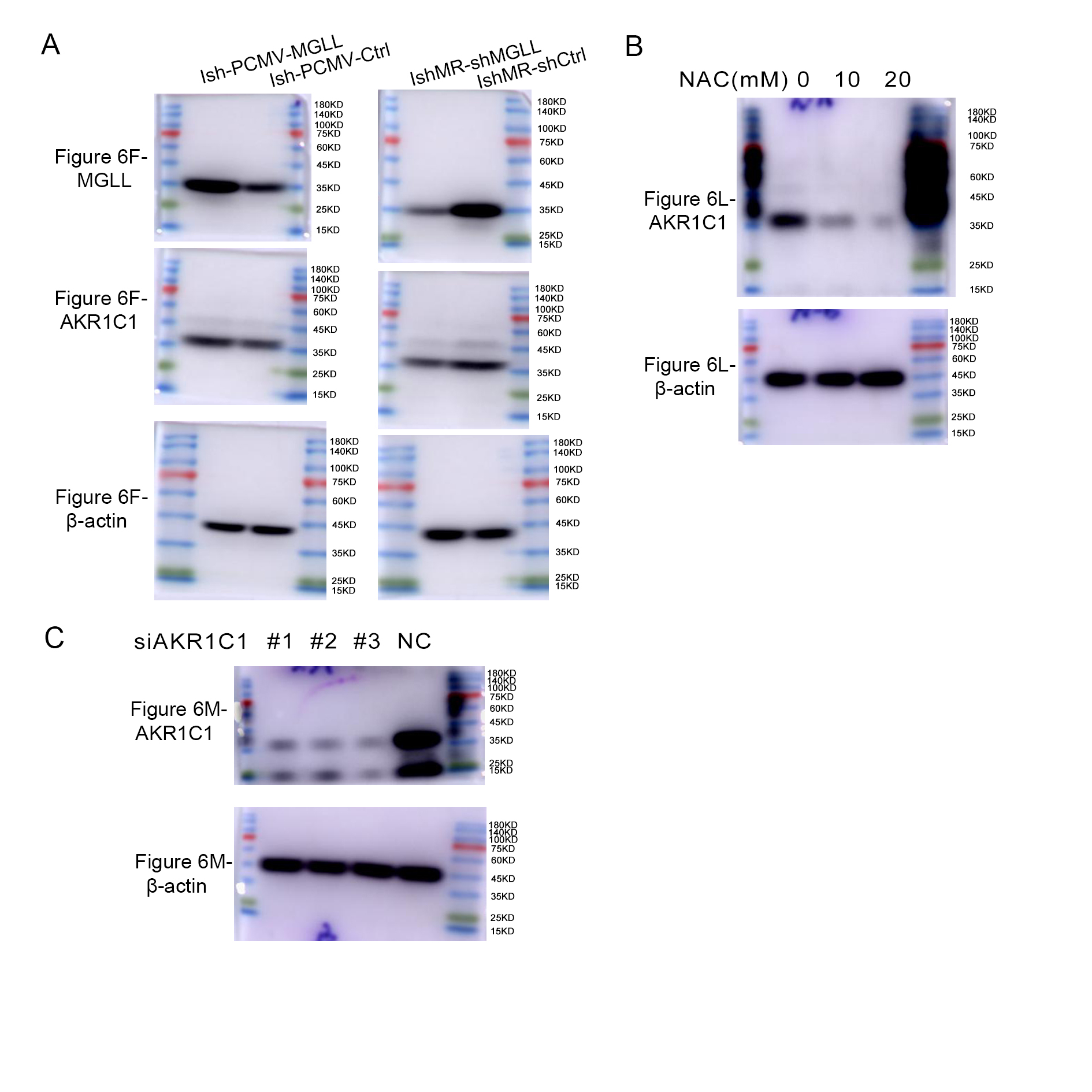

Supplement: Supplementary file 8 — Original data 6 [file 41419_2022_5507_MOESM8_ESM.jpg]

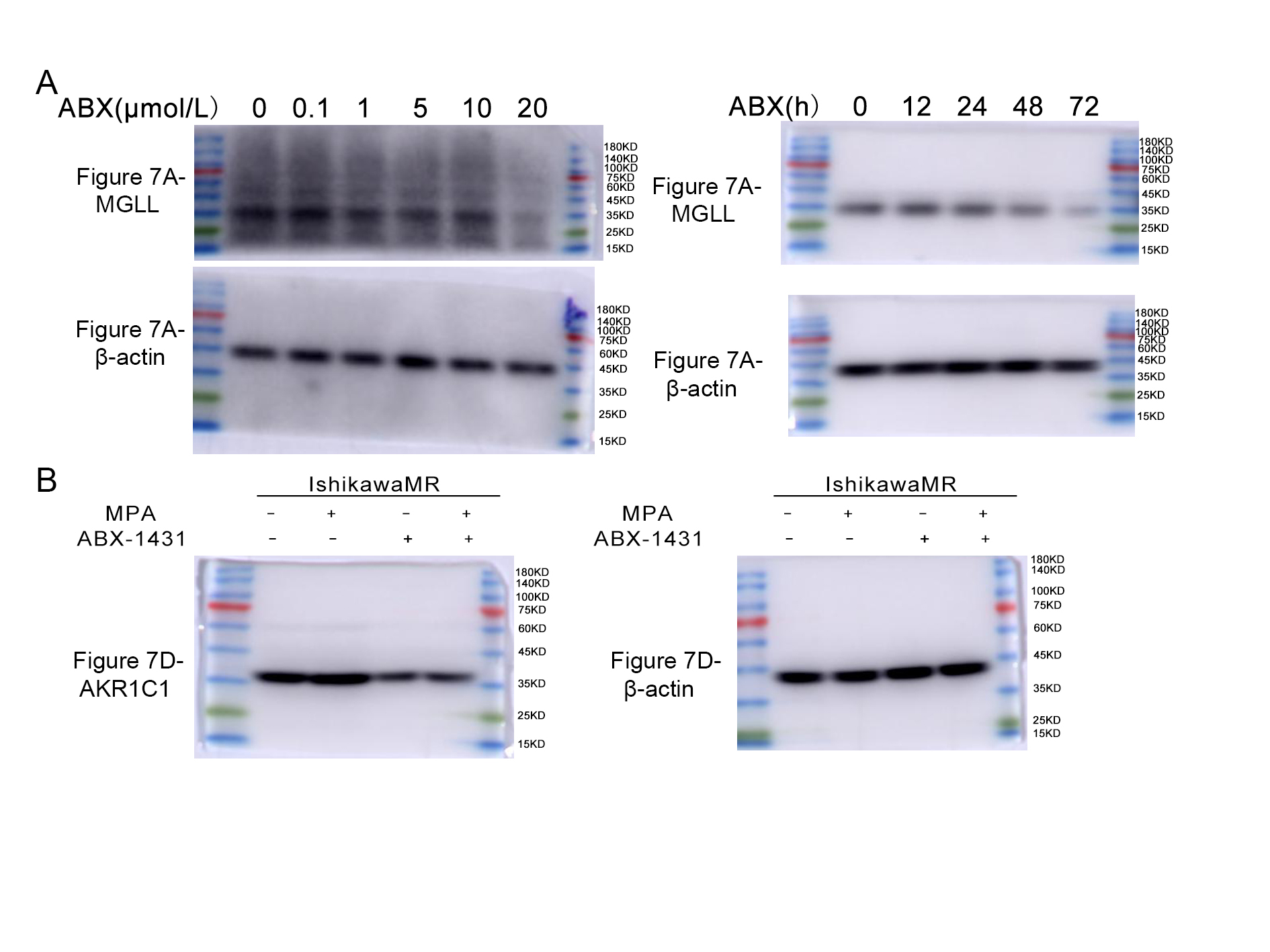

Supplement: Supplementary file 9 — Original data 7 [file 41419_2022_5507_MOESM9_ESM.jpg]
