# Supplementary material for: ABX-1431 inhibits the development of endometrial adenocarcinoma and reverses progesterone resistance by targeting MGLL
Source: Cell Death Dis. 2022 Dec 23;13(12):1067. doi: 10.1038/s41419-022-05507-z (PMC9780207; doi:10.1038/s41419-022-05507-z)
Supplement: Supplementary file 10 — Author Contribution Statement [file 41419_2022_5507_MOESM10_ESM.docx]

**Contribution to preparation of manuscript:**

Xiaohong Ma, Min Xia, Jie Jiang and Chunping Qiu conceived and designed experiments; Xiaohong Ma and Min Xia conducted the experiments; Jie Jiang and Chunping Qiu reviewed and revised the manuscript; Xiaohong Ma, Min Xia and Lina Wei collected tissue samples and clinical data; Xiaohong Ma, Min Xia and Kui Guo analyzed the data and wrote the manuscript. All authors read and approved the final manuscript.

**Detailed preparation of figures:**

In Figure 1-7, Xiaohong Ma and Min Xia generated the clinical data and analyzed the data; Min Xia and Kui Guo generated the immunohistochemistry data and labelled the image; Xiaohong Ma and Chunping Qiu generated the Edu and Western blotting data; Xiaohong Ma and Yao Liu analyzed the bioinformatics data; Min Xia and Lina Wei conducted the animal experiments; Xiaohong Ma and Min Xia generated the remaining data and assembled the figures.
